# Supplementary material for: Direct activation of HSF1 by macromolecular crowding and misfolded proteins
Source: PLoS One. 2024 Nov 4;19(11):e0312524. doi: 10.1371/journal.pone.0312524 (PMC11534217; doi:10.1371/journal.pone.0312524)

3-22: LPVGPGAAGPSNVPAFLTKL (#1)

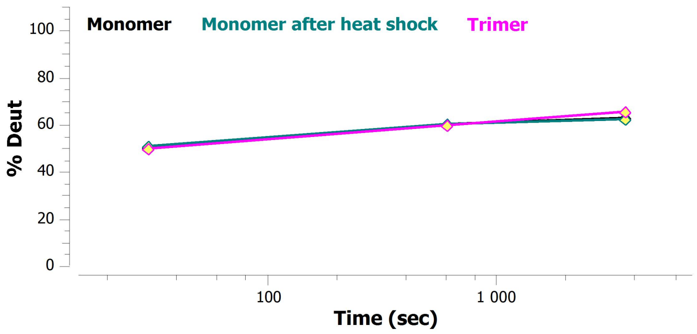

23-34: WTLVSDPDTDAL (#2)

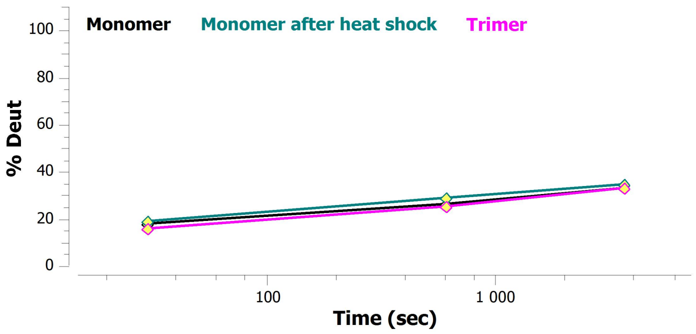

26-47: VSDPDTDALICWSPSGNSFHVF (#3)

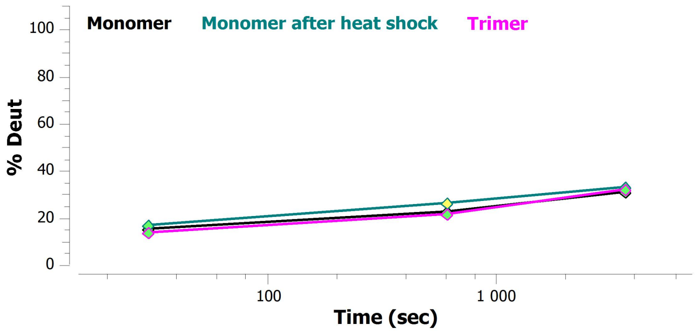

35-44: ICWSPSGNSF (#4)

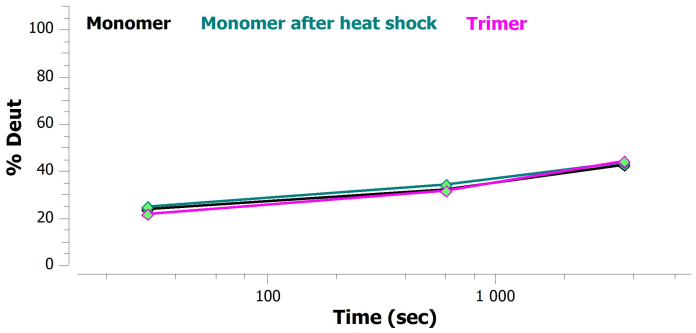

35-47: ICWSPSGNSFHVF (#5)

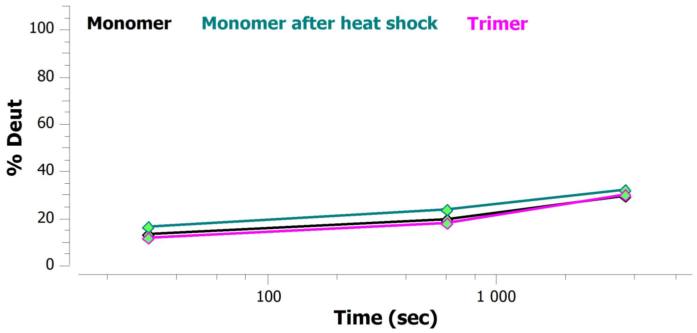

37-47: WSPSGNSFHVF (#6)

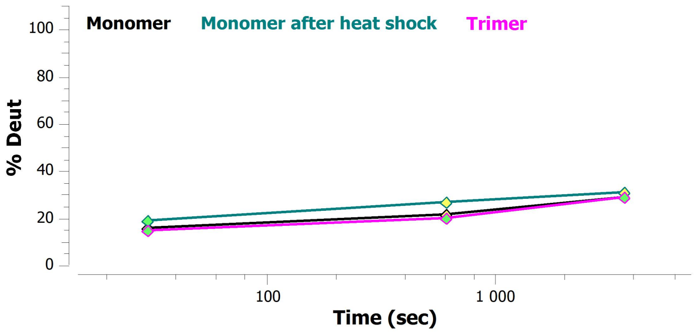

48-65: DQGQFAKEVLPKYFKHNN (#7)

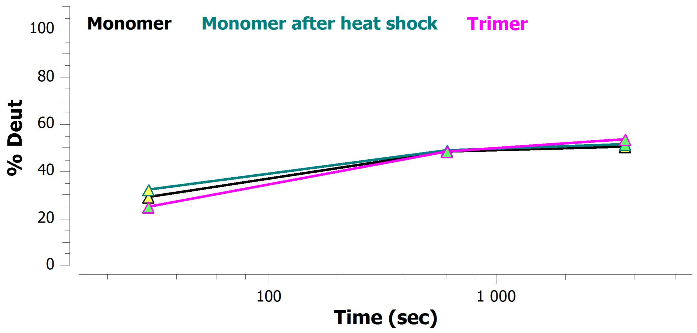

55-65: EVLPKYFKHNN (#8)

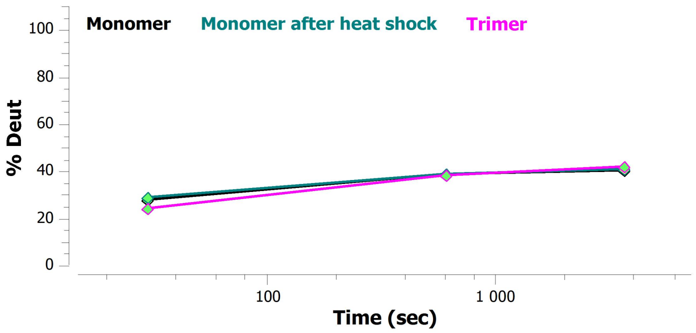

55-66: EVLPKYFKHNNM (#9)

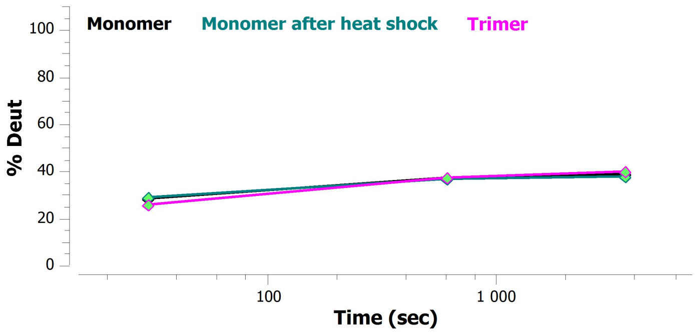

55-67: EVLPKYFKHNNMA (#10)

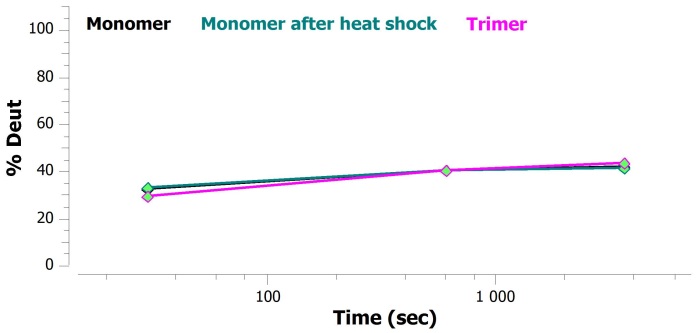

67-73: ASFVRQL (#11)

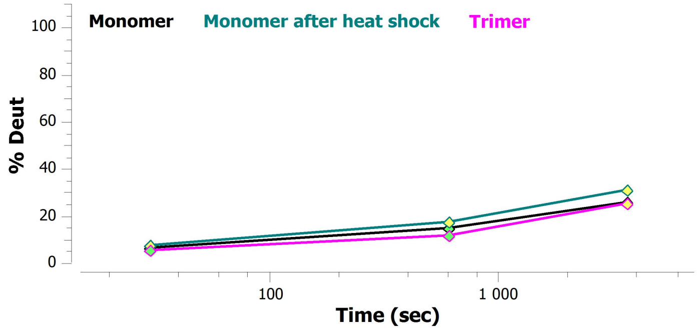

74-80: NMYGFRK (#12)

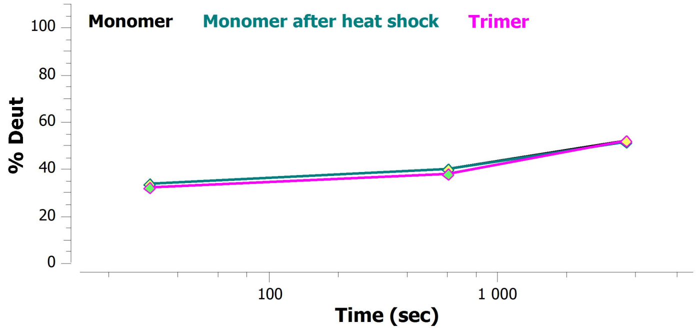

81-95: VVHIEQGGLVKPERD (#13)

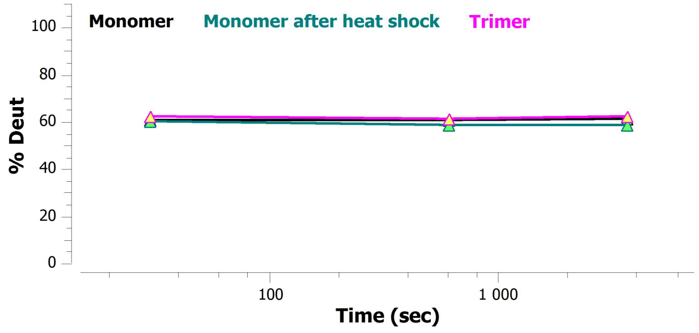

81-96: VVHIEQGGLVKPERDD (#14)

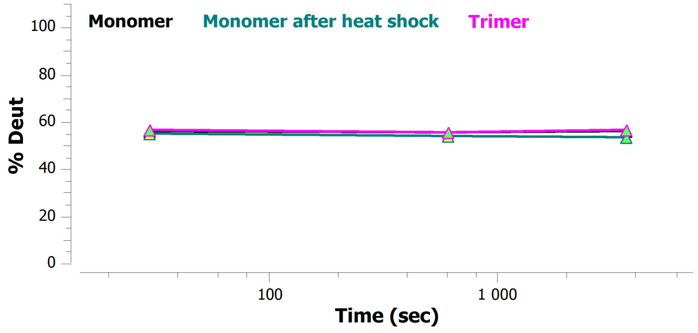

81-104: VVHIEQGGLVKPERDDTEFQHPCF (#15)

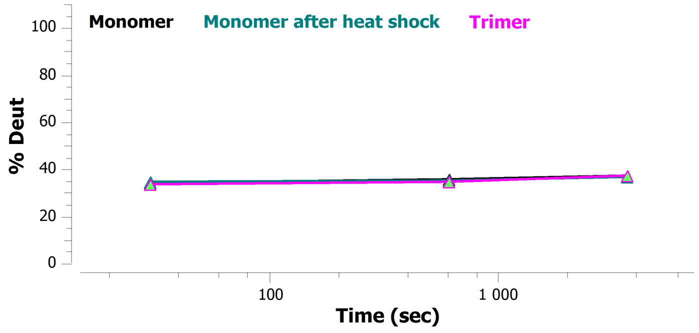

96-104: DTEFQHPCF (#16)

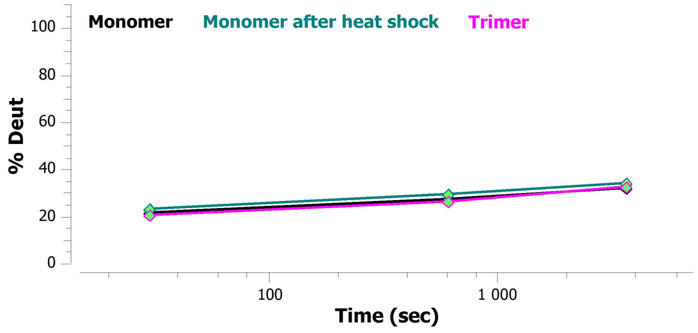

96-111: DTEFQHPCFLRGQEQL (#17)

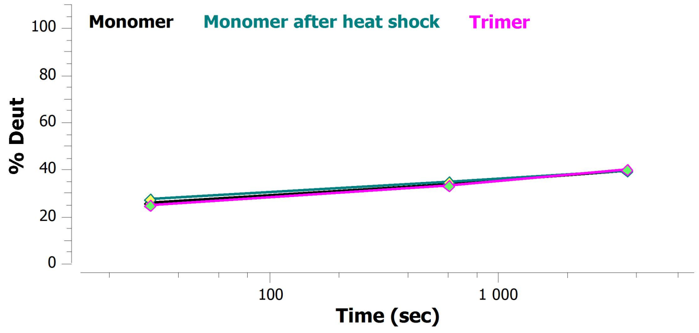

97-104: TEFQHPCF (#18)

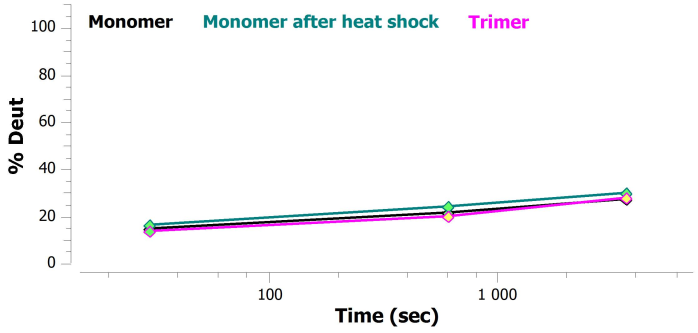

112-128: LENIKRKVTSVSTLKSE (#19)

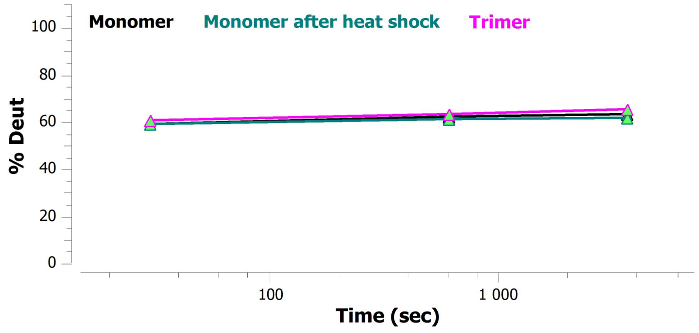

112-131: LENIKRKVTSVSTLKSEDIK (#20)

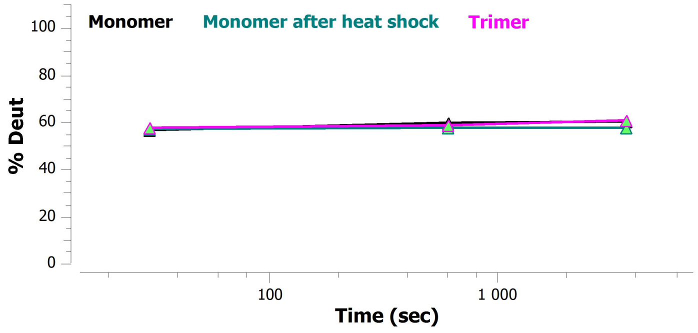

112-135: LENIKRKVTSVSTLKSEDIKIRQD (#21)

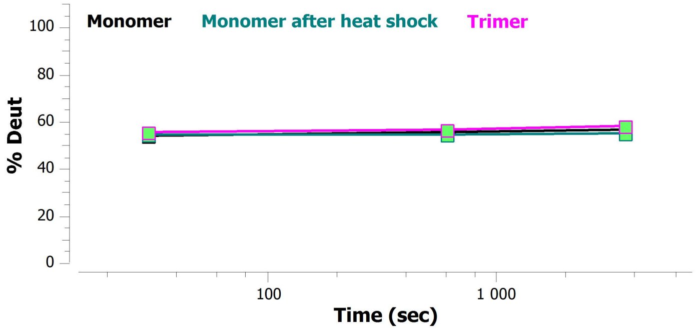

115-131: IKRKVTSVSTLKSEDIK (#22)

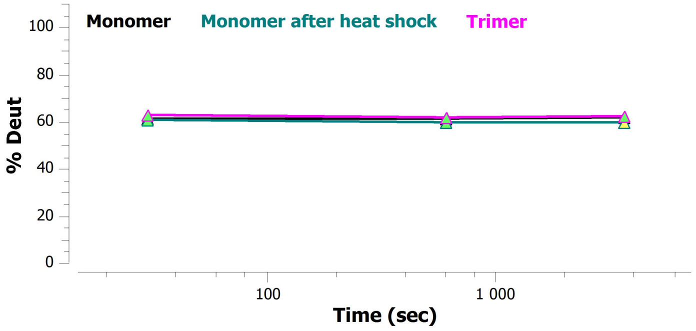

119-131: VTSVSTLKSEDIK (#23)

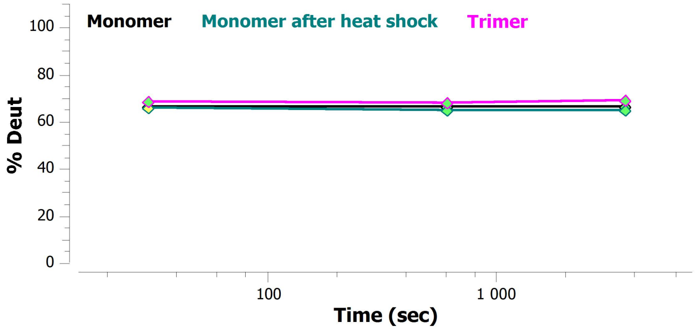

132-154: IRQDSVTKLLTDVQLMKGKQECM (#24)

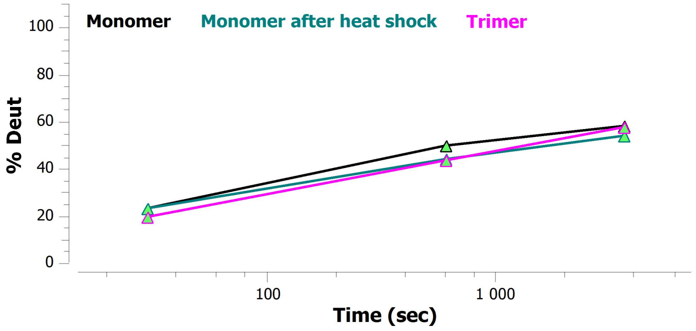

136-146: SVTKLLTDVQL (#25)

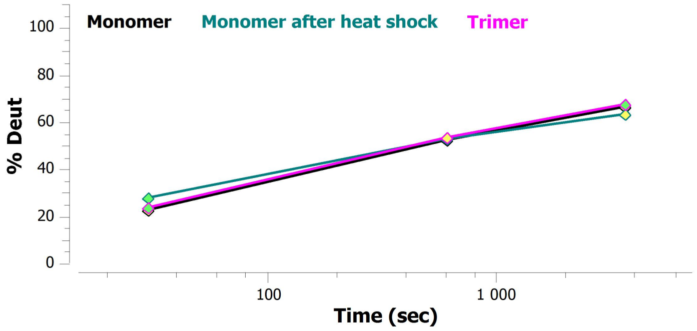

136-152: SVTKLLTDVQLMKGKQE (#26)

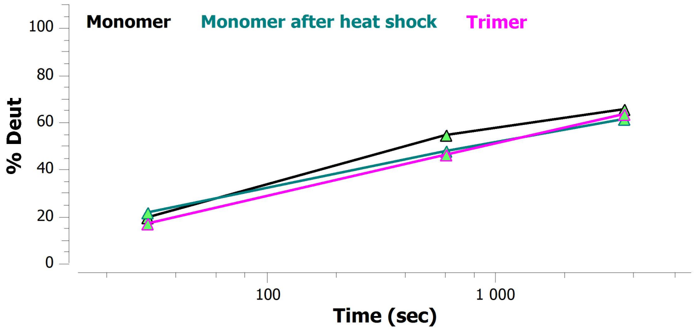

136-154: SVTKLLTDVQLMKGKQECM (#27)

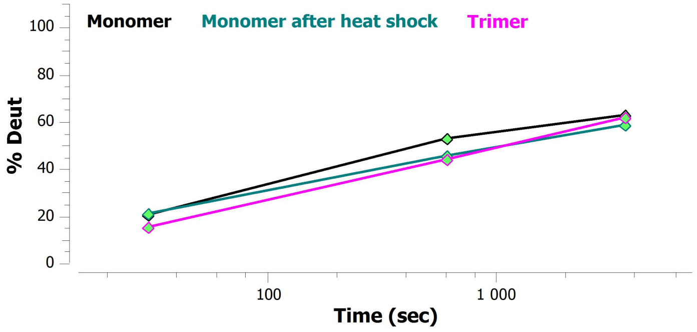

158-168: LLAMKHENEAL (#28)

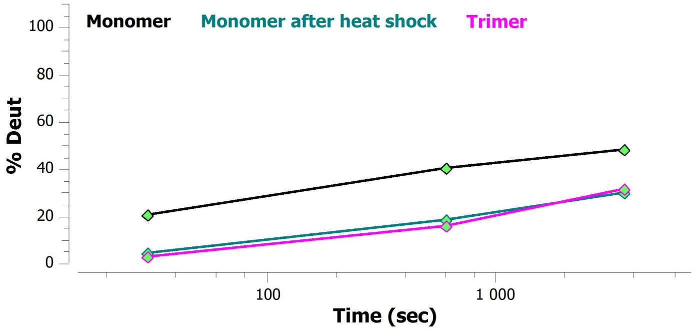

159-168: LAMKHENEAL (#29)

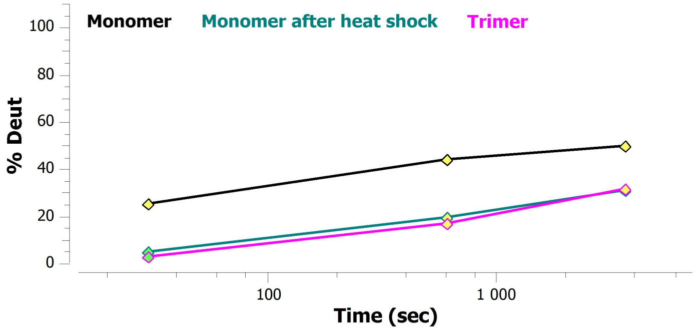

169-178: WREVASLRQK (#30)

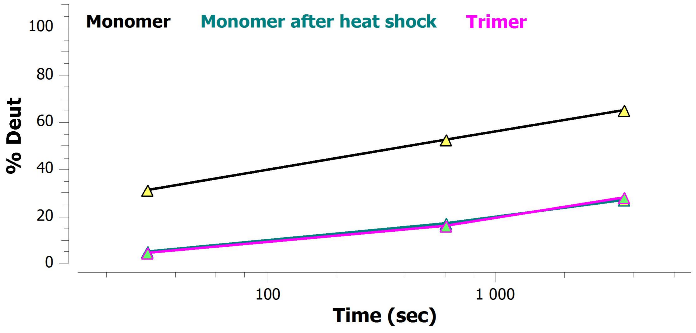

169-184: WREVASLRQKHAQQQK (#31)

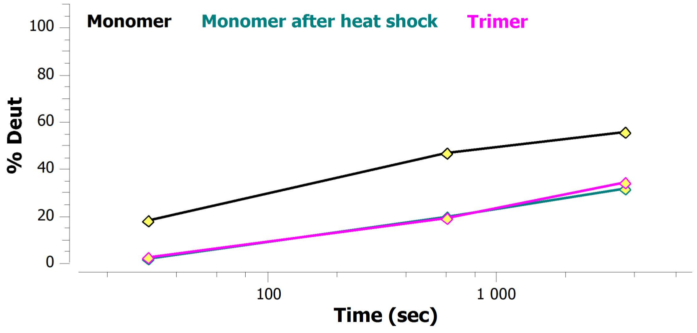

169-193: WREVASLRQKHAQQQKVVNKLIQFL (#32)

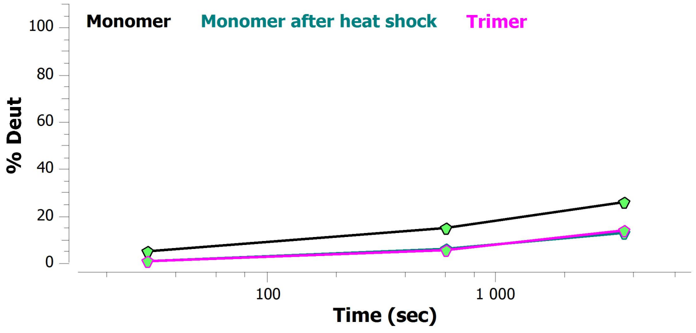

169-195: WREVASLRQKHAQQQKVVNKLIQFLIS (#33)

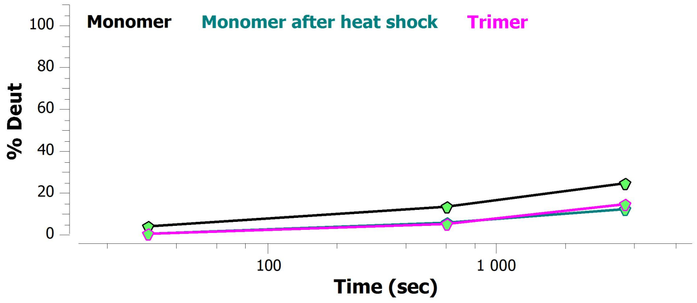

172-184: VASLRQKHAQQQK (#34)

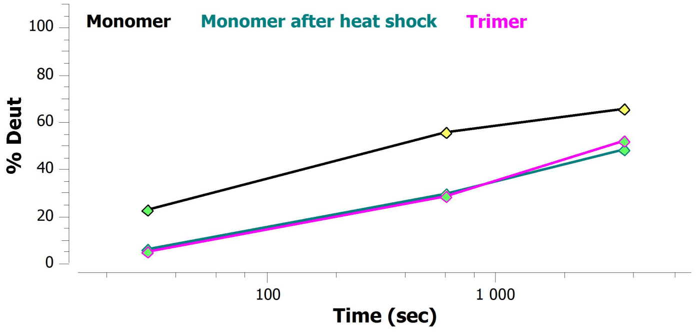

172-193: VASLRQKHAQQQKVVNKLIQFL (#35)

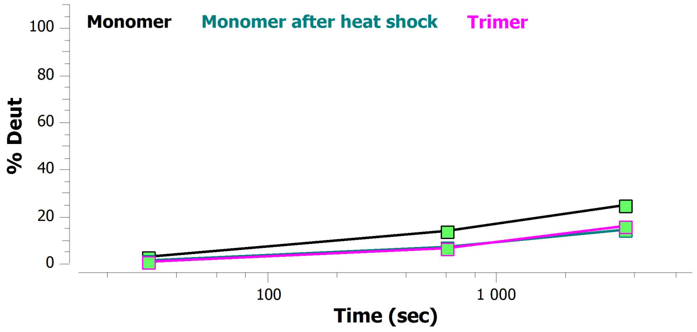

172-195: VASLRQKHAQQQKVVNKLIQFLIS (#36)

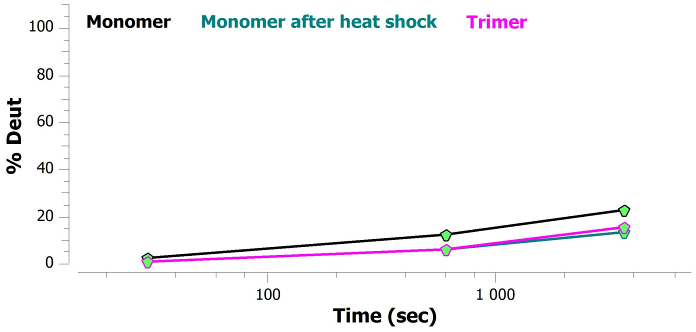

174-195: SLRQKHAQQQKVVNKLIQFLIS (#37)

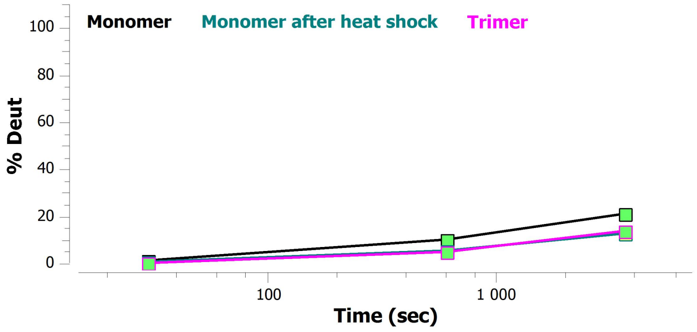

179-193: HAQQQKVVNKLIQFL (#38)

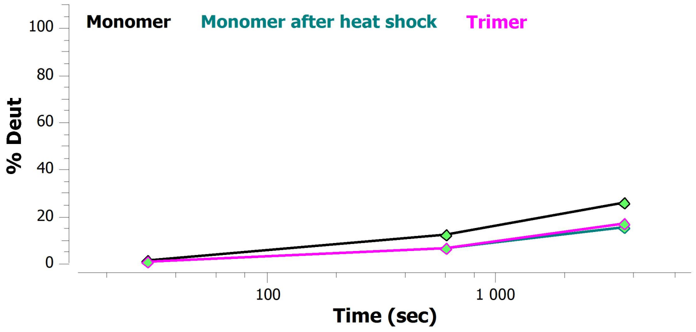

185-193: VVNKLIQFL (#39)

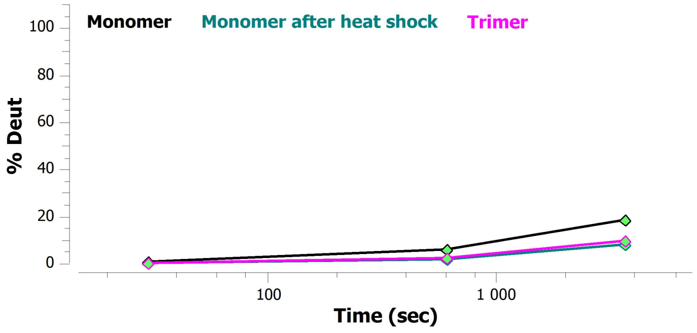

185-195: VVNKLIQFLIS (#40)

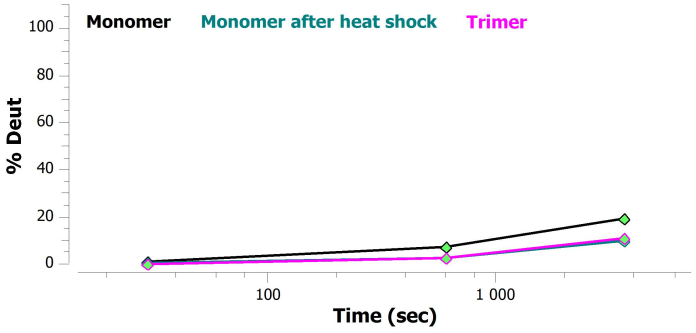

185-196: VVNKLIQFLISL (#41)

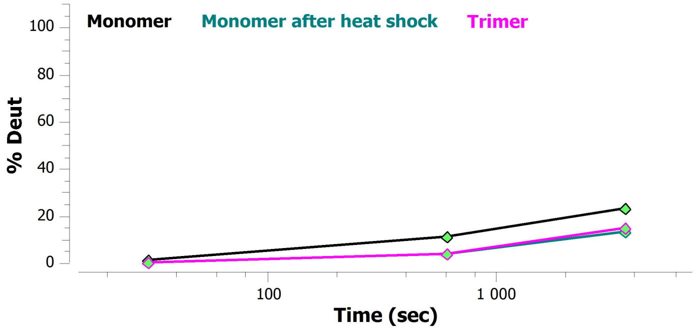

196-229: LVQSNRILGVKRKIPLMLNDSGSAHSMPKYSRQF (#42)

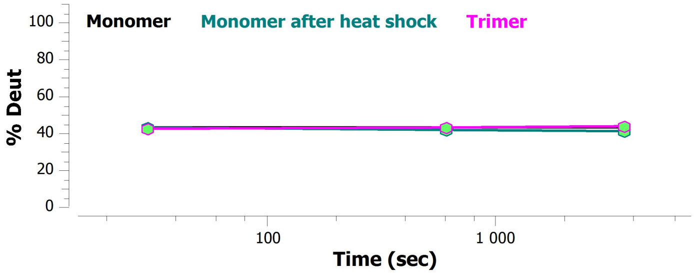

197-213: VQSNRILGVKRKIPLML (#43)

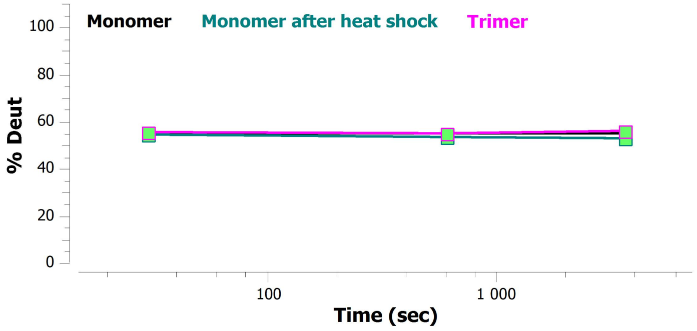

204-213: GVKRKIPLML (#44)

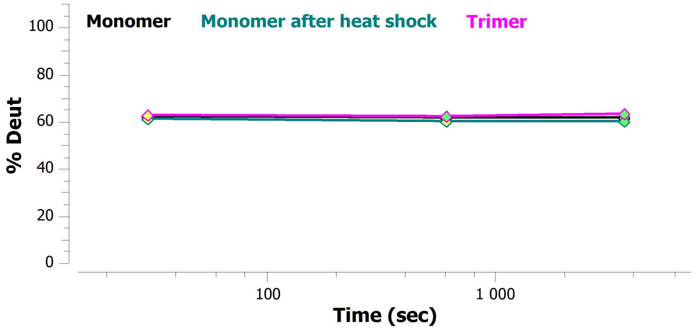

204-229: GVKRKIPLMLNDSGSAHSMPKYSRQF (#45)

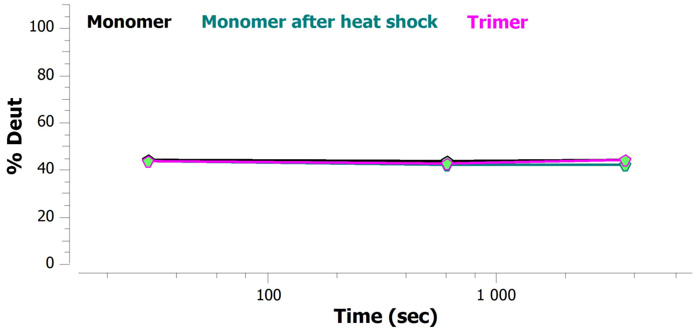

204-231: GVKRKIPLMLNDSGSAHSMPKYSRQFSL (#46)

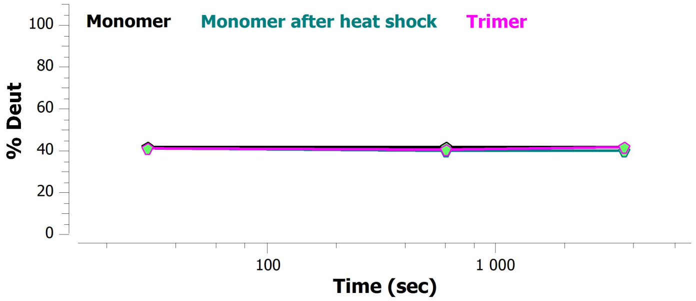

214-229: NDSGSAHSMPKYSRQF (#47)

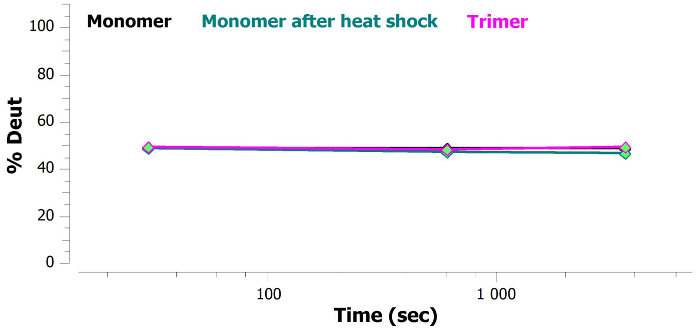

228-252: QFSLEHVHGSGPYSAPSPAYSSSSL (#48)

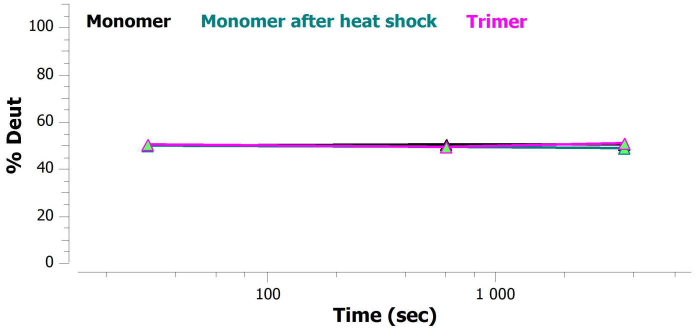

229-252: FSLEHVHGSGPYSAPSPAYSSSSL (#49)

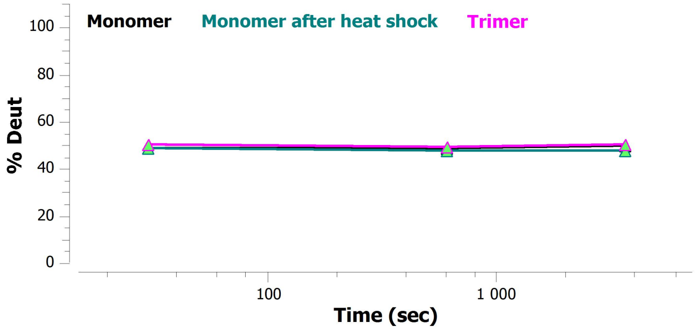

230-252: SLEHVHGSGPYSAPSPAYSSSSL (#50)

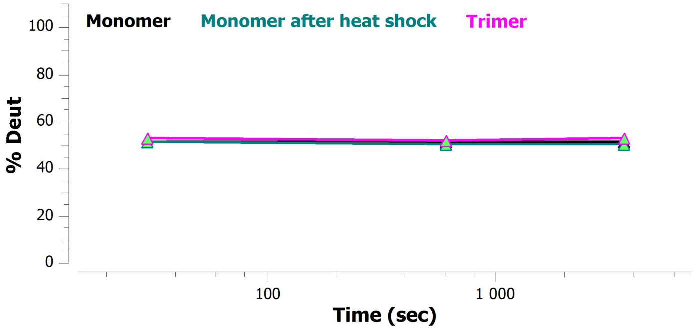

232-252: EHVHGSGPYSAPSPAYSSSSL (#51)

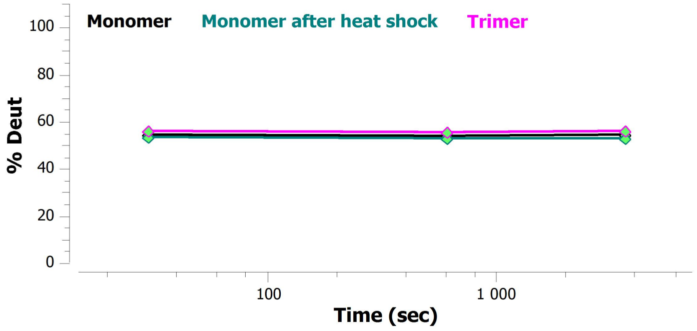

253-296: APDAVASSGPIISDITELAPASPMASPGGSIDERPLSSSPLV (#52)

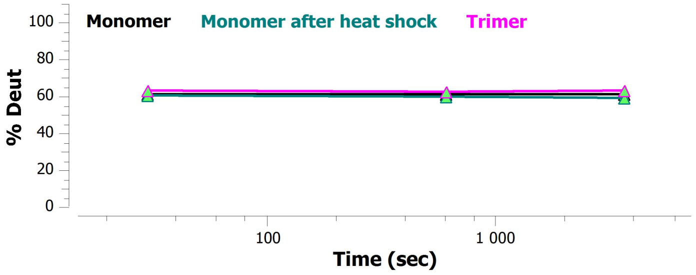

267-296: DITELAPASPMASPGGSIDERPLSSSPLVR (#53)

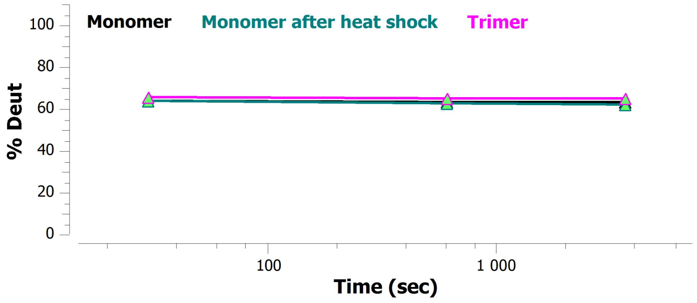

297-330: VKEEPPSPPQSPRVEEASPGRPSSVDTLLSPTAL (#54)

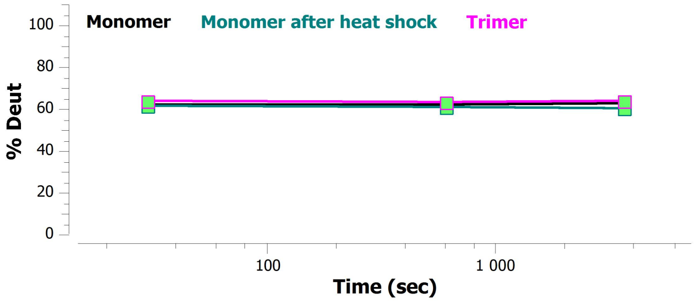

297-332: VKEEPPSPPQSPRVEEASPGRPSSVDTLLSPTALID (#55)

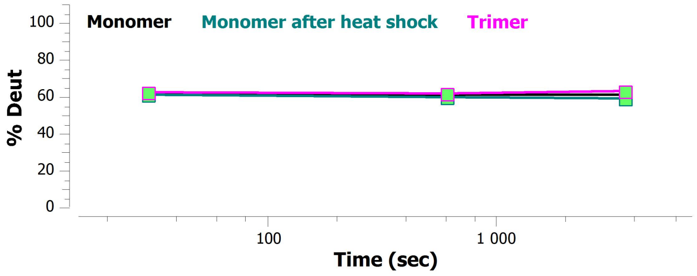

312-332: EASPGRPSSVDTLLSPTALID (#56)

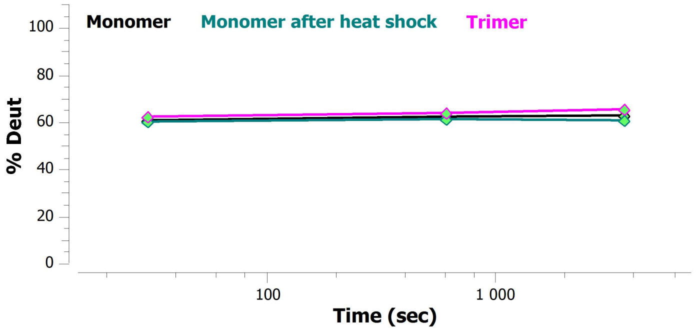

333-372: SILRESEPAPASVTALTDARGHTDTEGRPPSPPPTSTPEK (#57)

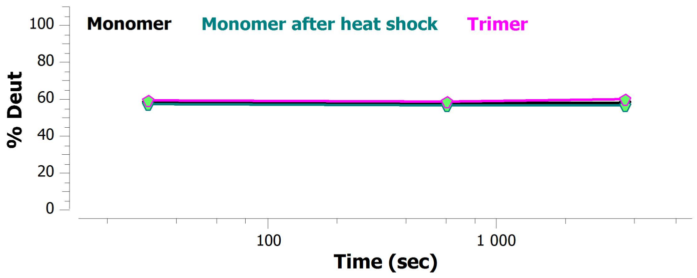

333-374: SILRESEPAPASVTALTDARGHTDTEGRPPSPPPTSTPEKCI (#58)

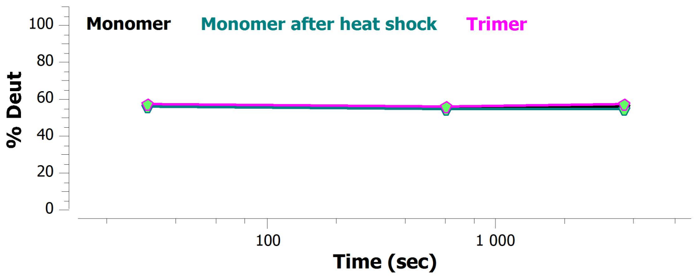

336-347: RESEPAPASVTA (#59)

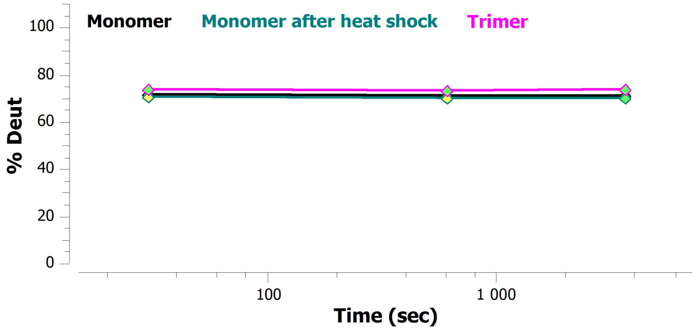

336-372: RESEPAPASVTALTDARGHTDTEGRPPSPPPTSTPEK (#60)

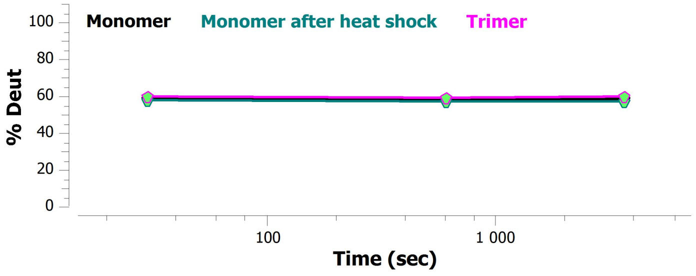

336-374: RESEPAPASVTALTDARGHTDTEGRPPSPPPTSTPEKCL (#61)

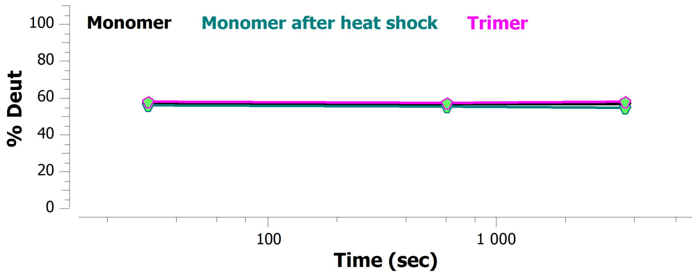

348-374: LTDARGHTDTEGRPPSPPPTSTPEKCL (#62)

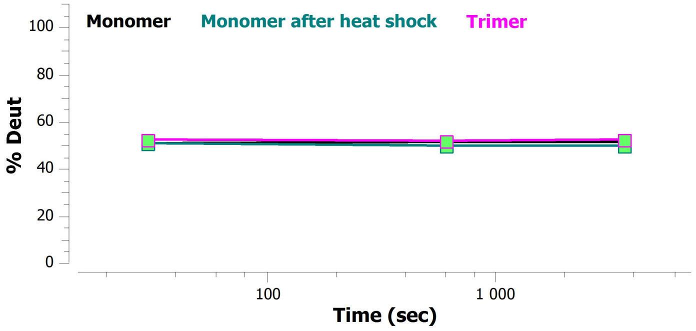

351-374: ARGHTDTEGRPPSPPPTSTPEKCL (#63)

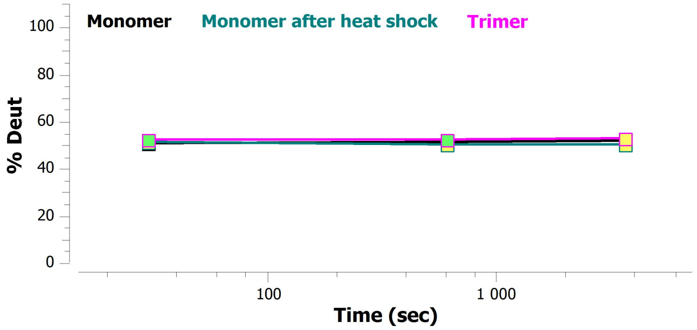

357-374: TEGRPPSPPPTSTPEKCL (#64)

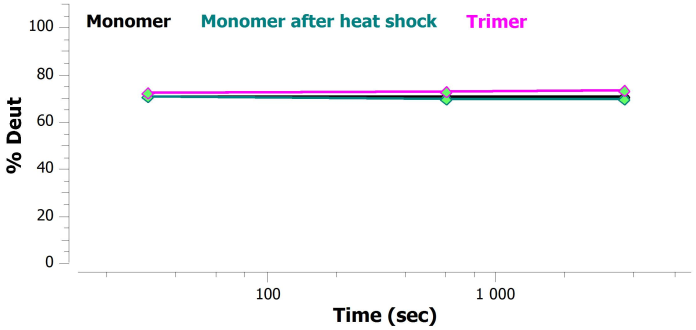

380-389: DKNELSDHLD (#65)

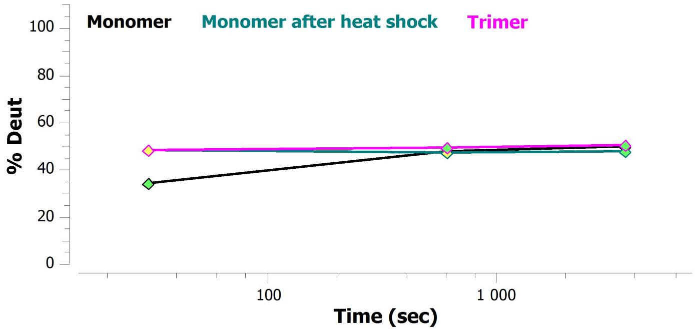

382-389: NELSDHLD (#66)

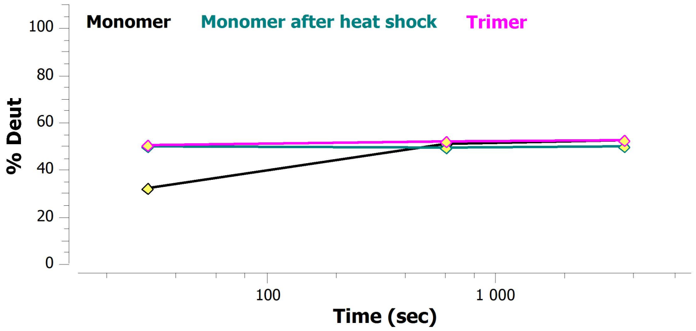

382-391: NELSDHLDAM (#67)

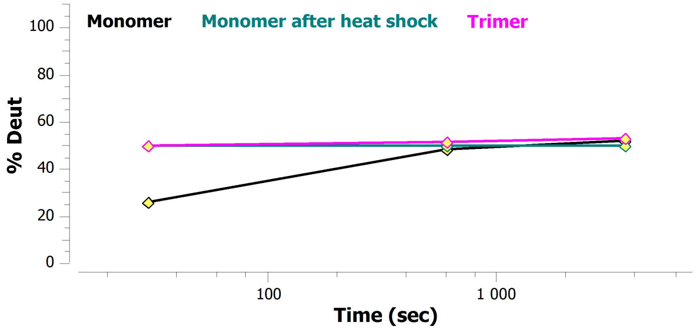

382-398: NELSDHLDAMDSNLDNL (#68)

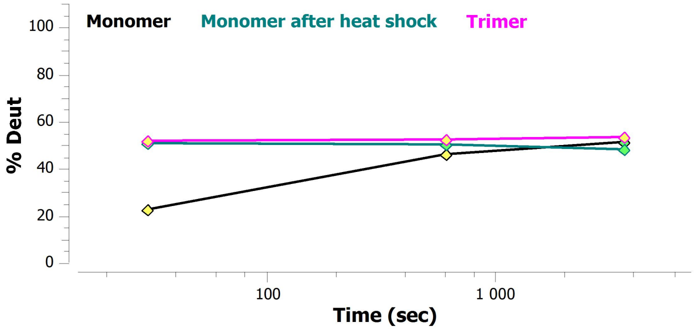

389-407: DAMDSNLDNLQTMLSSHGF (#69)

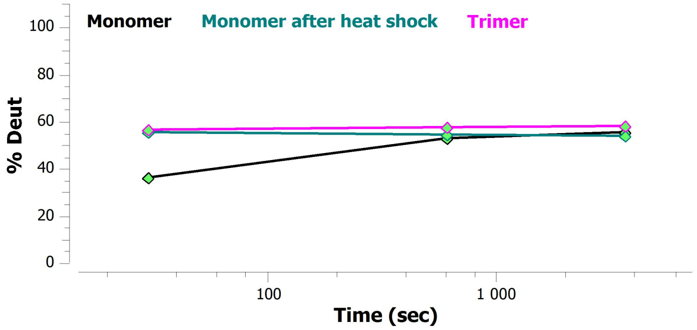

390-407: AMDSNLDNLQTMLSSHGF (#70)

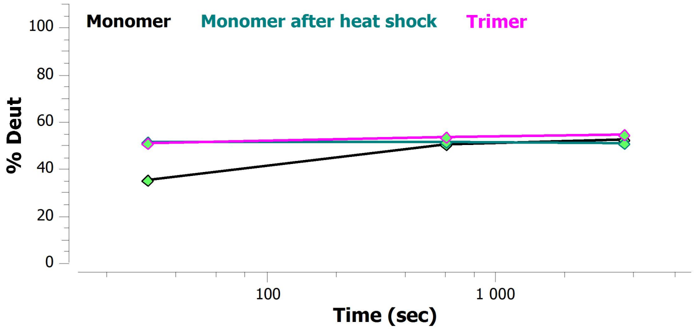

392-407: DSNLDNLQTMLSSHGF (#71)

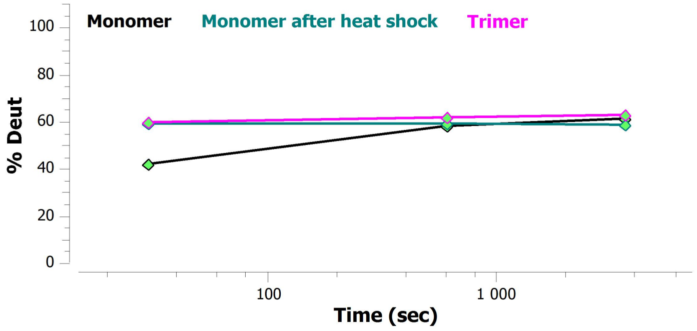

396-407: DNLQTMLSSHGF (#72)

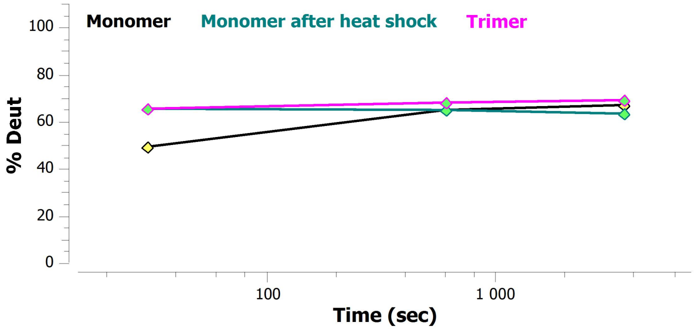

399-407: QTMLSSHGF (#73)

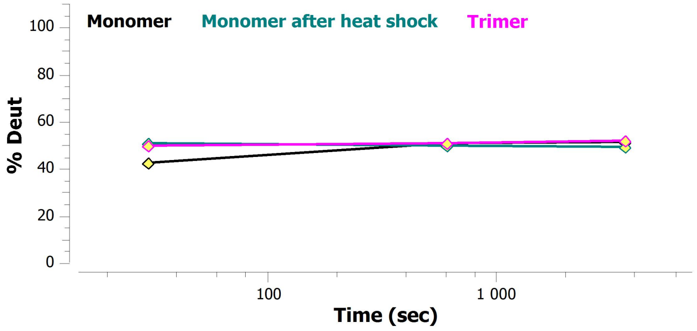

416-432: DLFSPSVTVPDMSLPDL (#74)

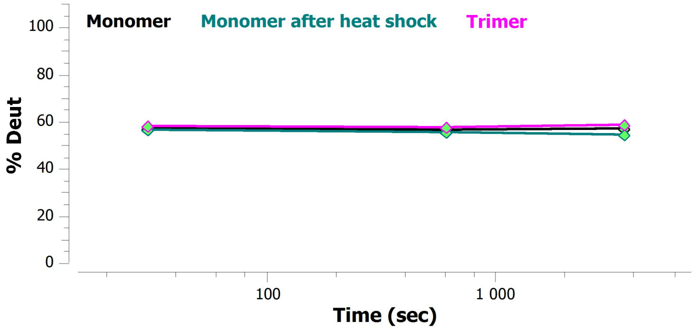

416-436: DLFSPSVTVPDMSLPDLDSSL (#75)

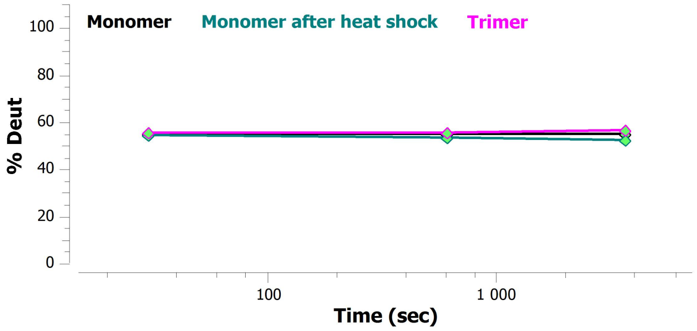

417-436: LFSPSVTVPDMSLPDLDSSL (#76)

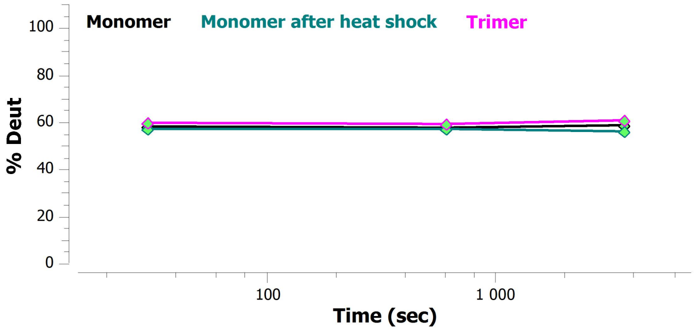

418-433: FSPSVTVPDMSLPDLD (#77)

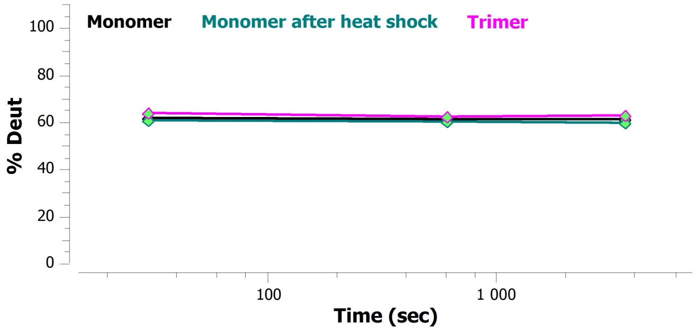

418-436: FSPSVTVPDMSLPDLDSSL (#78)

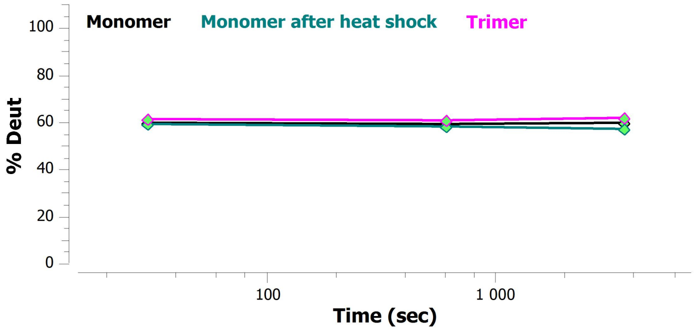

437-465: ASIQELLSPQEPPRPPEAENSSPDSGKQL (#79)

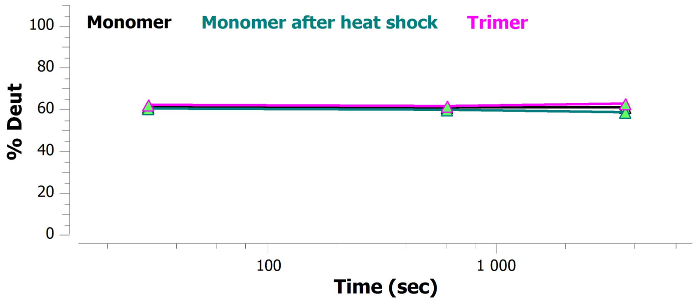

437-475: ASIQELLSPQEPPRPPEAENSSPDSGKQLVHYTAQPLFL (#80)

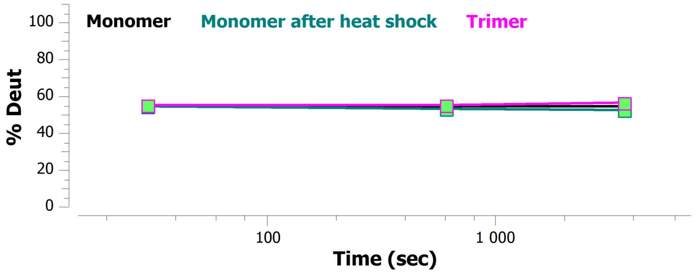

437-476: ASIQELLSPQEPPRPPEAENSSPDSGKQLVHYTAQPLFLL (#81)

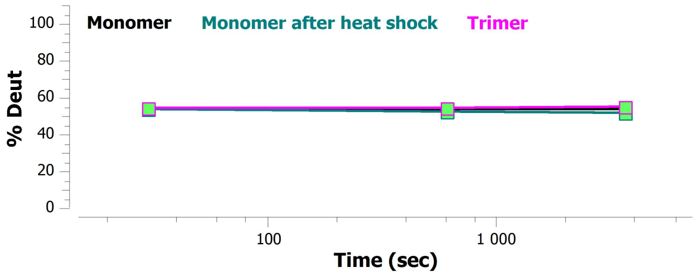

442-475: LLSPQEPPRPPEAENSSPDSGKQLVHYTAQPLFL (#82)

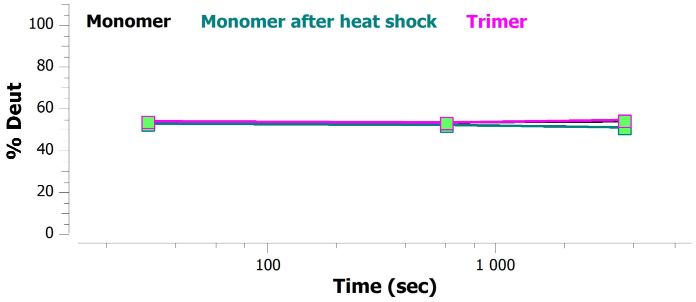

443-465: LSPQEPPRPPEAENSSPDSGKQL (#83)

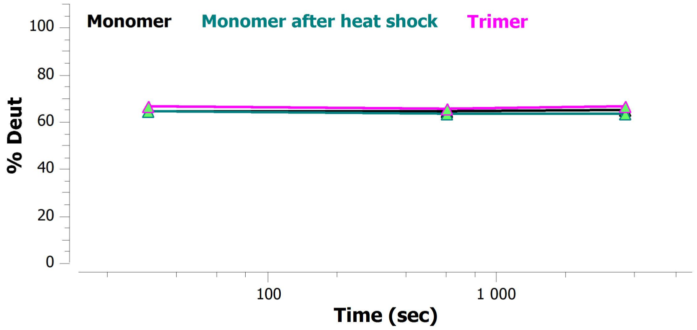

443-475: LSPQEPPRPPEAENSSPDSGKQLVHYTAQPLFL (#84)

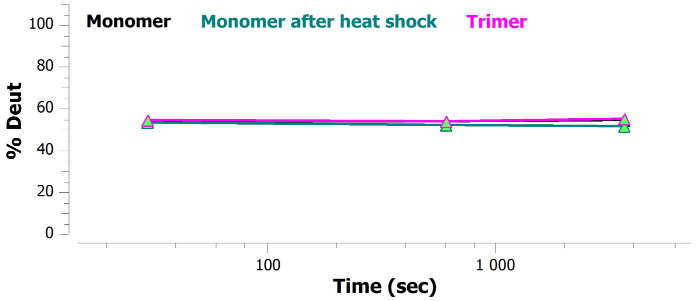

443-482: LSPQEPPRPPEAENSSPDSGKQLVHYTAQPLFLLDPGSVD (#85)

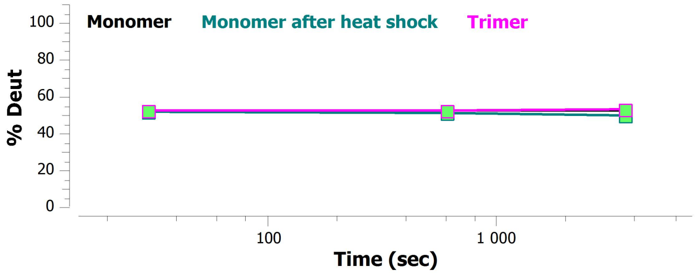

444-475: SPQEPPRPPEAENSSPDSGKQLVHYTAQPLFL (#86)

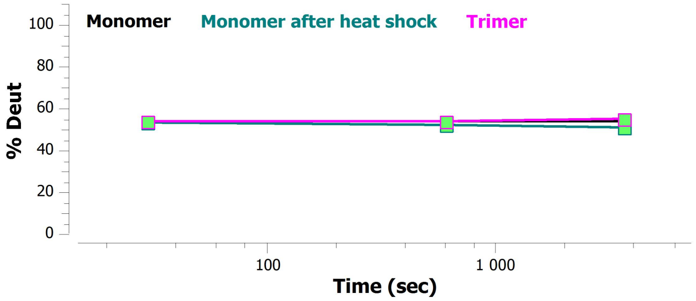

466-475: VHYTAQPLFL (#87)

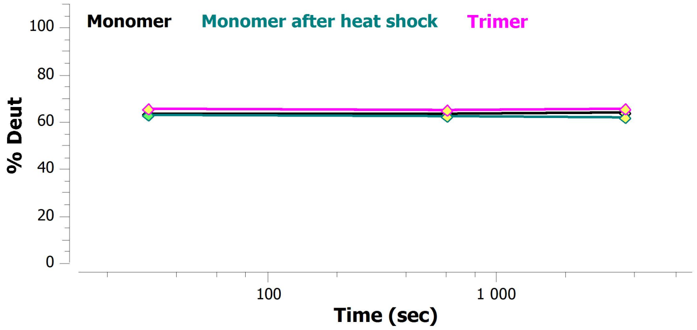

466-482: VHYTAQPLFLLDPGSVD (#88)

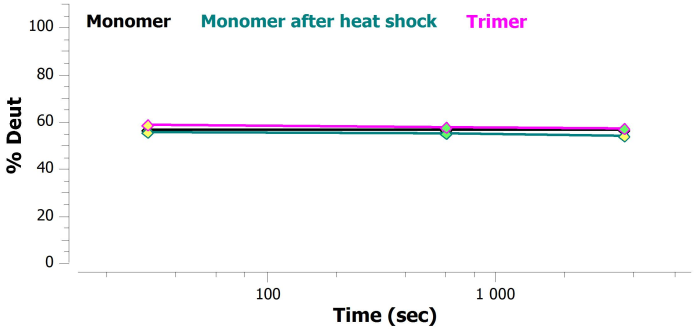

476-491: LDPGSVDTGSNDLPVL (#89)

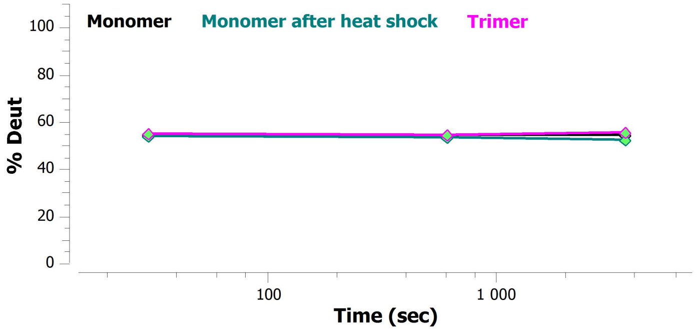

492-529: FELGEGSYFSEGDGFAEDPTISLLTGSEPPKAKDPTVS (#90)

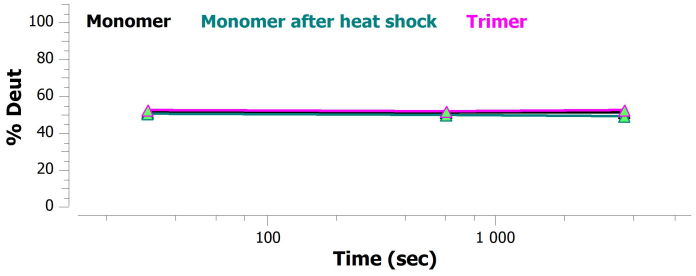

500-515: FSEGDGFAEDPTISLL (#91)

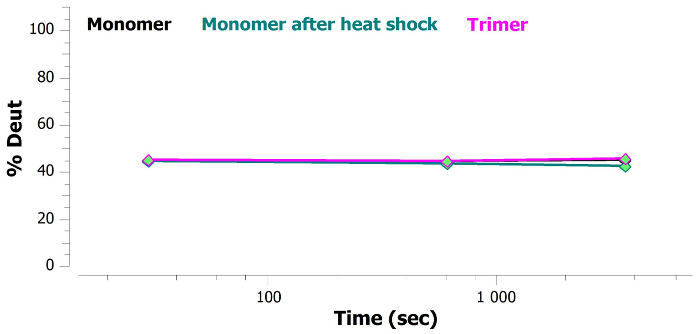

507-515: AEDPTISLL (#92)

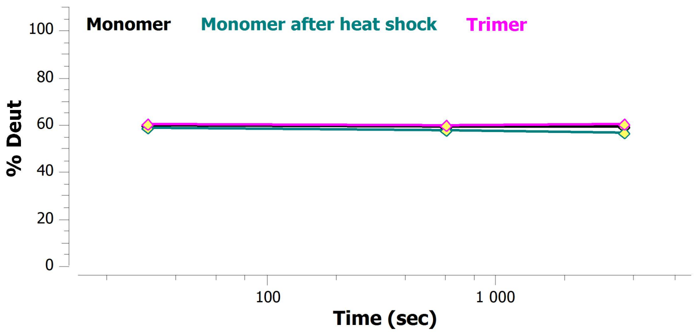

515-529: LTGSEPPKAKDPTVS (#93)

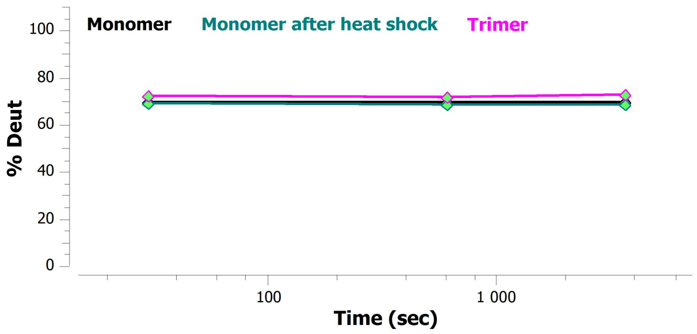

516-529: TGSEPPKAKDPTVS (#94)

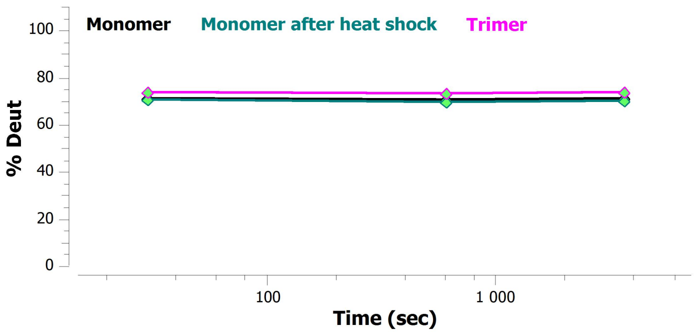

Supplement: S1 Fig — A list of peptide uptake plots for individual peptides of HSF1. The data compare deuteration levels over time for HSF1 monomer, HSF1 monomer after 42°C heat shock, and HSF1 trimer. (PDF) [file pone.0312524.s001.pdf]
