# Supplementary figures and images for: Direct activation of HSF1 by macromolecular crowding and misfolded proteins
Source: PLoS One. 2024 Nov 4;19(11):e0312524. doi: 10.1371/journal.pone.0312524 (PMC11534217; doi:10.1371/journal.pone.0312524)

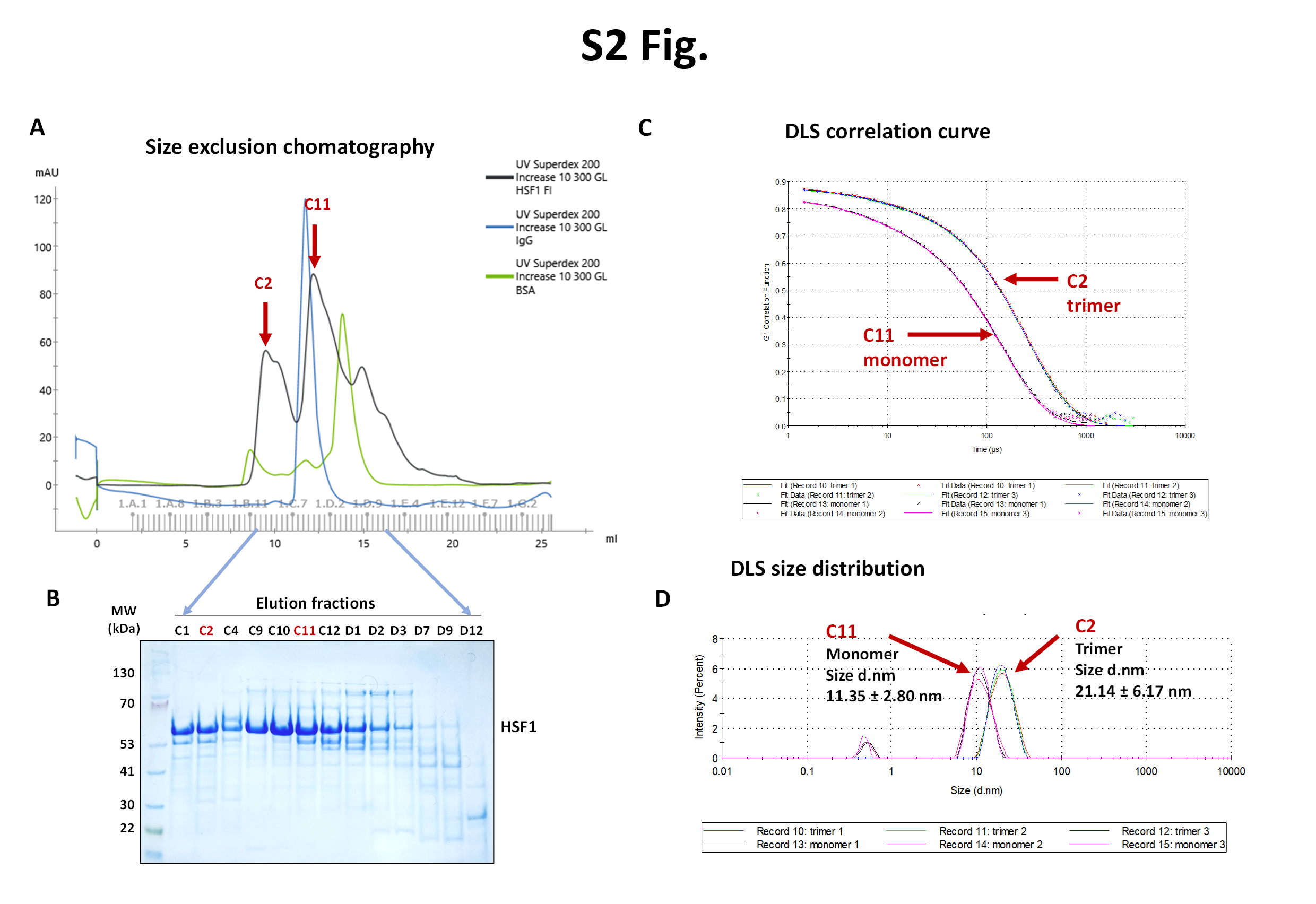

Supplement: S2 Fig — A: SEC chromatogram of HSF1 was compared to those of standard proteins, BSA (green) and IgG (blue), each loaded at 1 mg/ml. SEC was conducted under identical conditions for HSF1, BSA, and IgG. B: SEC fractions were analyzed by SDS-PAGE followed by Coomassie staining. C: Correlation curves from dynamic light scattering (DLS) measurements are presented for HSF1 monomer (fraction C11) and trimer (fraction C2) samples. All measurements were conducted in triplicates at 25°C. D: Size distribution profiles obtained from DLS for HSF1 monomer (fraction C11) and trimer (fraction C2) samples. The size distribution data shows a median of 11.35 nm with a standard deviation of 2.802 nm for the monomer, and a median of 21.14 nm with a standard deviation of 6.169 nm for the trimer. (TIF) [file pone.0312524.s002.tif]

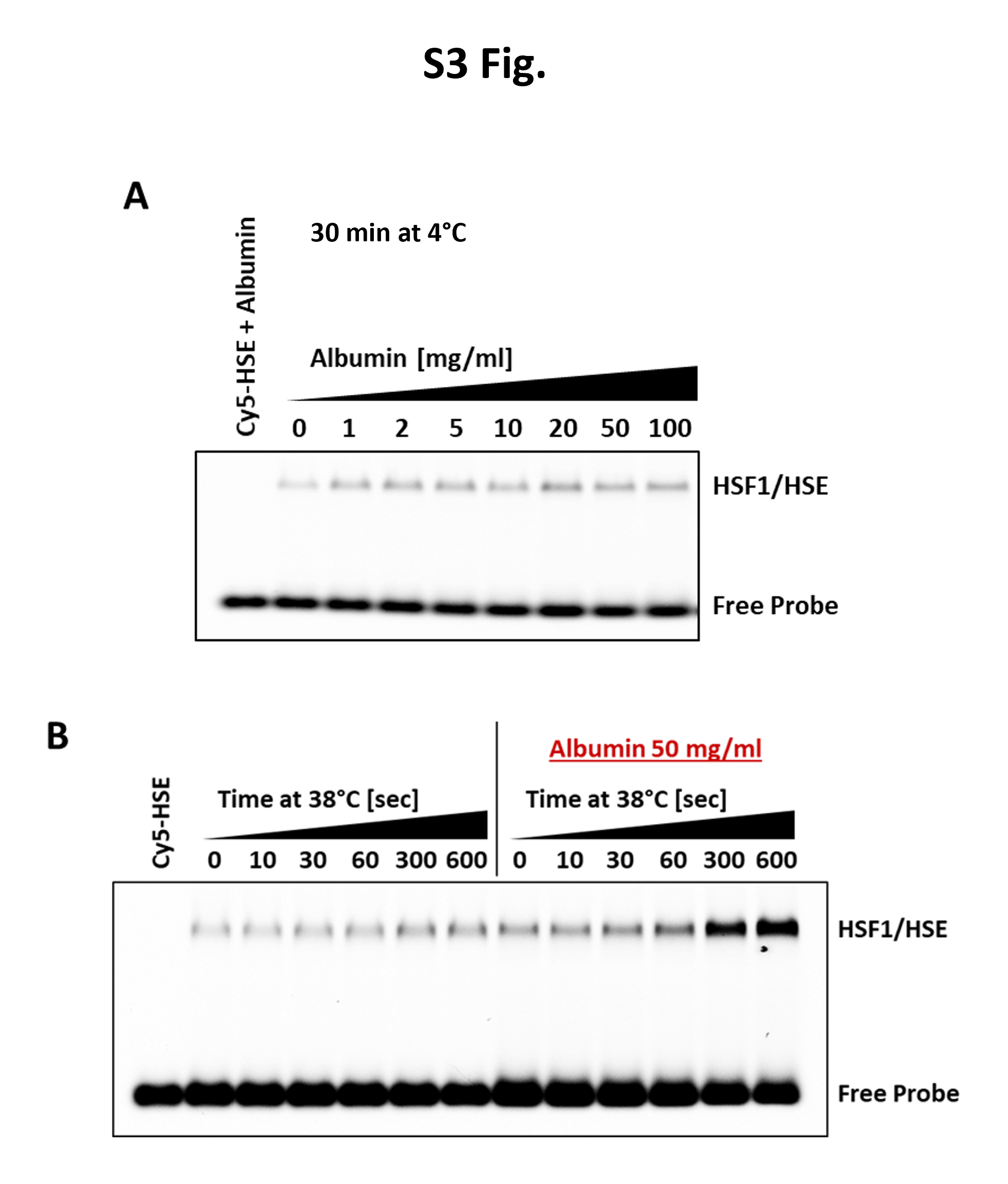

Supplement: S3 Fig — A: EMSA analysis showing the effect of increasing concentrations of BSA on HSF1 DNA binding at 4°C. HSF1 monomer (200 nM) and Cy5-HSE (100 nM) were incubated with BSA (0–100 mg/ml) for 30 minutes at 4°C. Results indicate that BSA does not impact DNA binding at this temperature. B: Comparison of HSF1 DNA binding with or without 50 mg/ml BSA at 38°C, across different incubation times (0–600 seconds). The data show that BSA influences HSF1 DNA binding kinetics at mildly elevated temperatures. (TIF) [file pone.0312524.s003.tif]

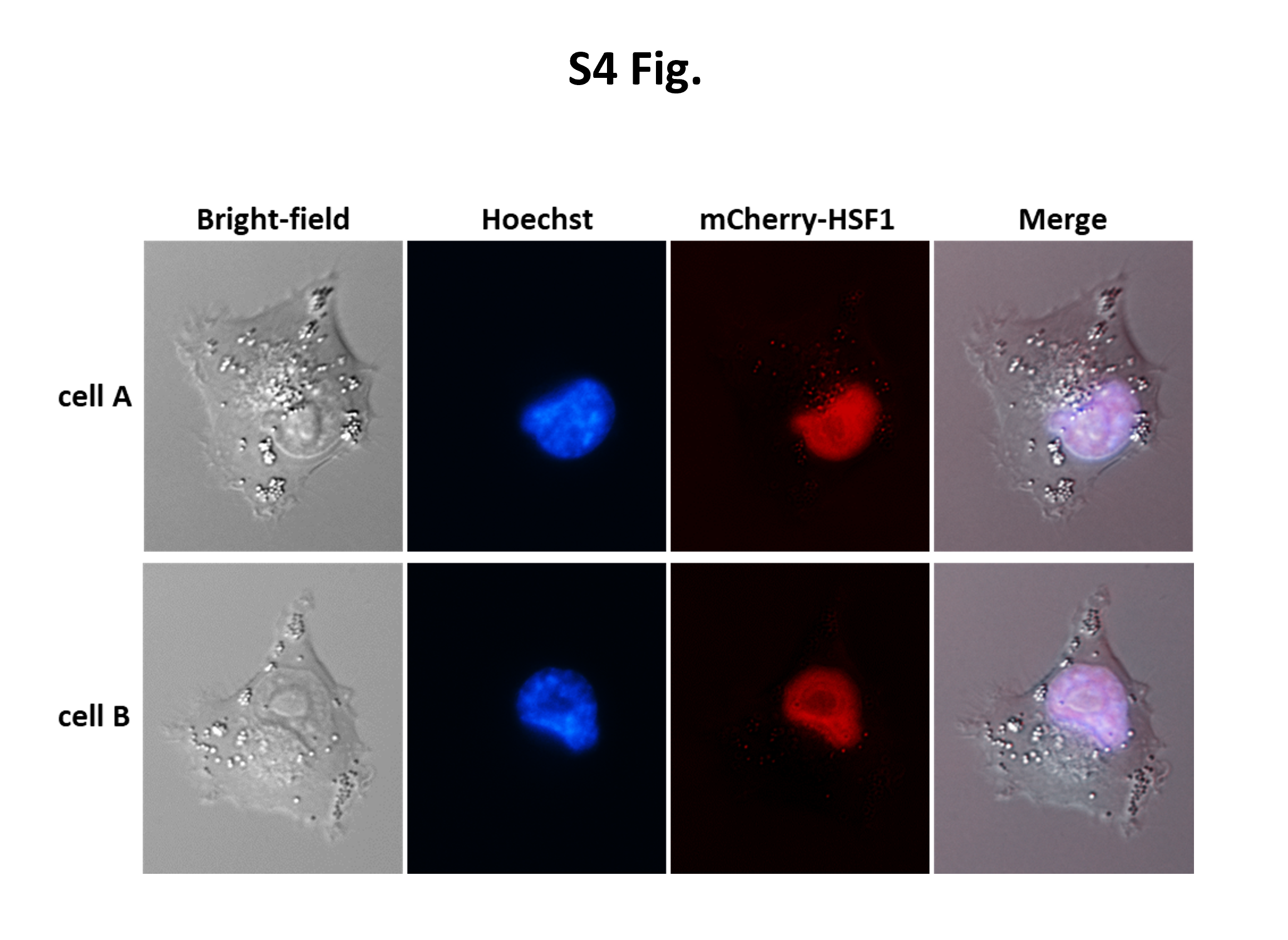

Supplement: S4 Fig — Microscopy images of H1299 cells expressing mCherry-HSF1. Cells were stained with Hoechst (1 μg/ml) for 15 minutes to visualize nuclei. The figure includes two representative cells A/B. The data confirm that under normal conditions, HSF1 exclusively localizes to the nucleus. (TIF) [file pone.0312524.s004.tif]

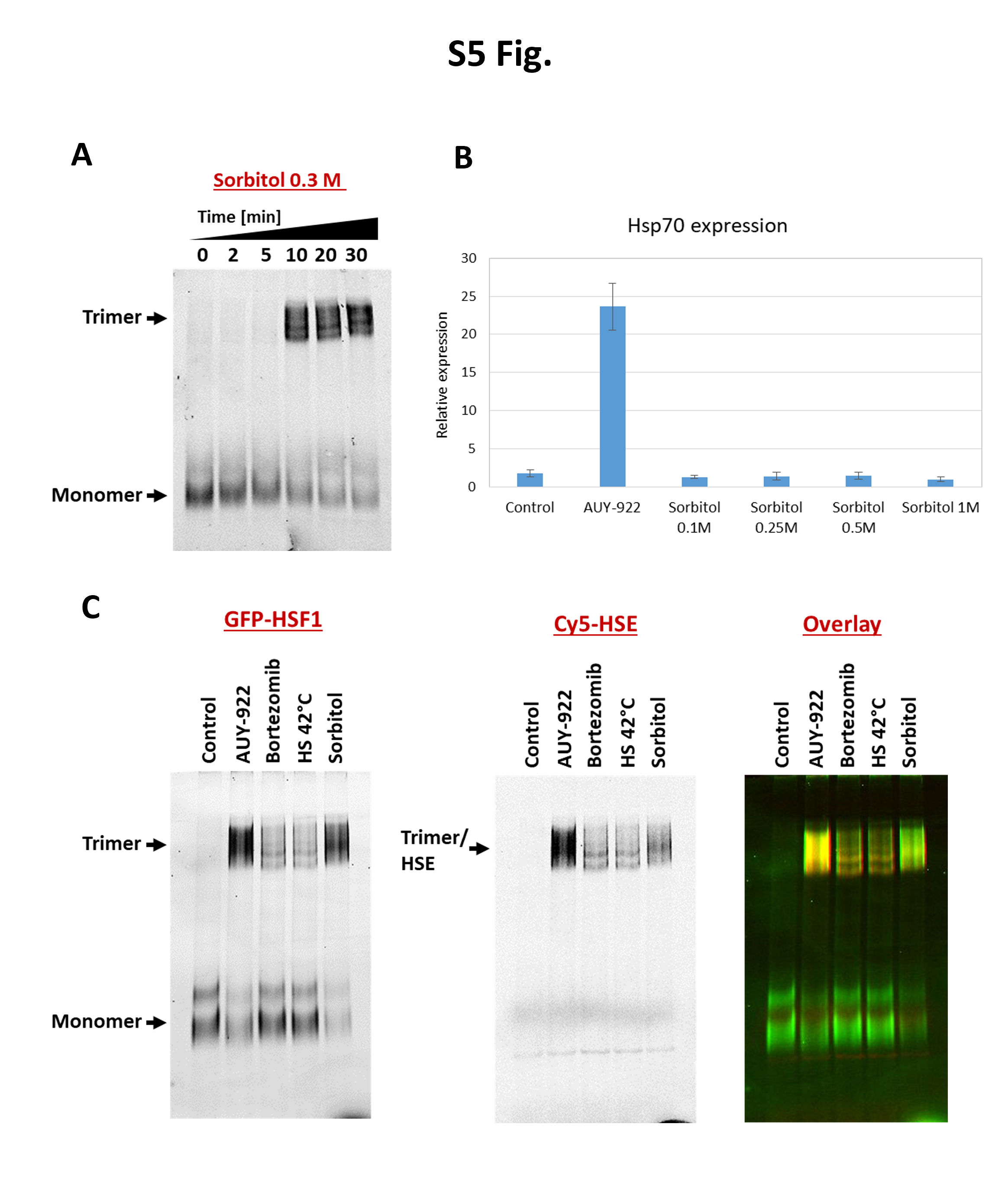

Supplement: S5 Fig — A: HR CNE showing the formation of HSF1 trimers in H1299 cells expressing mCherry-HSF1 after treatment with 0.3 M sorbitol for varying times (0–30 minutes). Trimer formation is observed after 10 minutes of sorbitol exposure. B: RT-qPCR analysis of Hsp70 expression in H1299 cells exposed to sorbitol. AUY-922 was used as a positive control. Although sorbitol leads to initiation of HSF1 activation, it does not result in the full activation of the heat shock response. C: EMSA employing HR CNE showing the effect of proteotoxic stress on HSF1 trimerization and DNA binding. H1299 cells expressing GFP-HSF1 were treated with 300 nM AUY-922, 300 nM bortezomib and heat shock 42°C for 1 hour, or 0.3 M sorbitol for 20 minutes. Cell lysates were incubated with 100 nM Cy5-HSE for 30 minutes at 4°C and analyzed by HR CNE and fluorescence imaging using a Typhoon FLA 9500. The native gel shows HSF1 trimerization under stress conditions, while EMSA confirms the ability of trimers to bind DNA. See S1 Raw images for raw data. (TIF) [file pone.0312524.s005.tif]
