# Supplementary material for: Direct activation of HSF1 by macromolecular crowding and misfolded proteins
Source: PLoS One. 2024 Nov 4;19(11):e0312524. doi: 10.1371/journal.pone.0312524 (PMC11534217; doi:10.1371/journal.pone.0312524)

Raw data to figure 2

Blue native electrophoresis of SEC fractions, followed by western blotting. Detection by anti HSF1 antibody c-5.

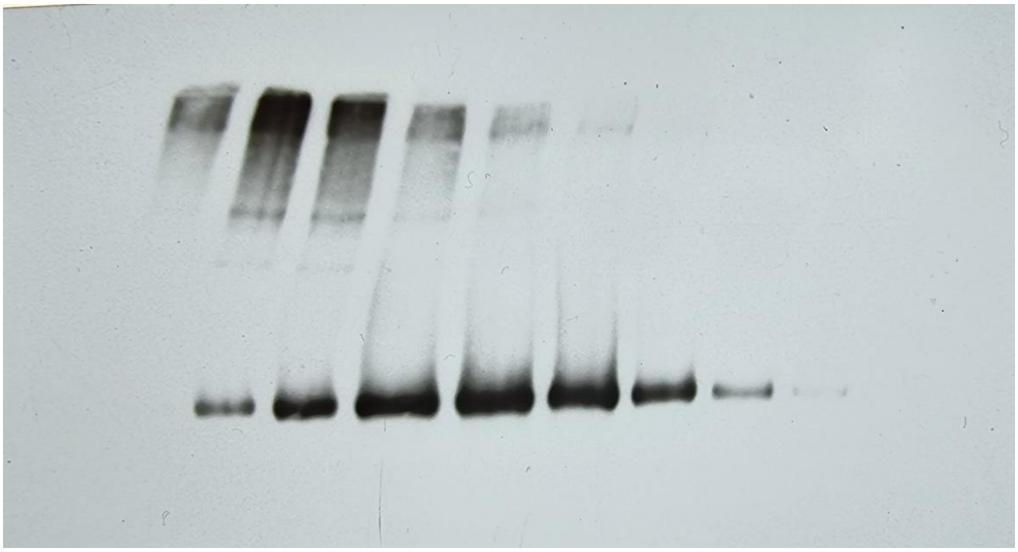

Raw data to figure 2

EMSA using SEC fractions and Cy5-HSE. Agarose gel with Cy5-HSE signal detected by Typhoon FLA 9500.

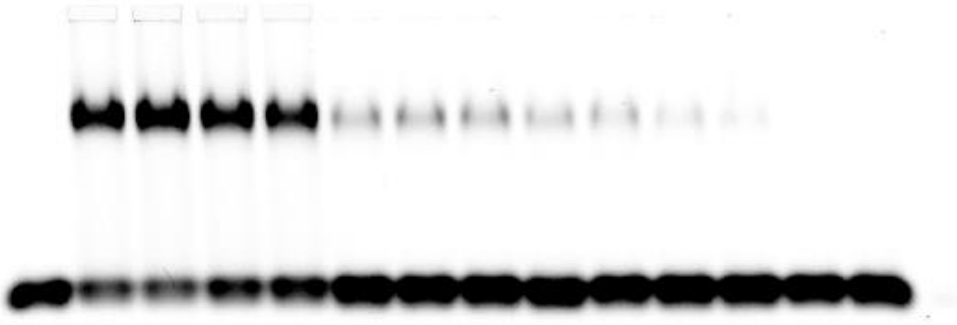

EMSA using SEC fractions and Cy5-HSE. Incubation at 40°C for 10 minutes. Agarose gel with Cy5-HSE signal detected by Typhoon FLA 9500.

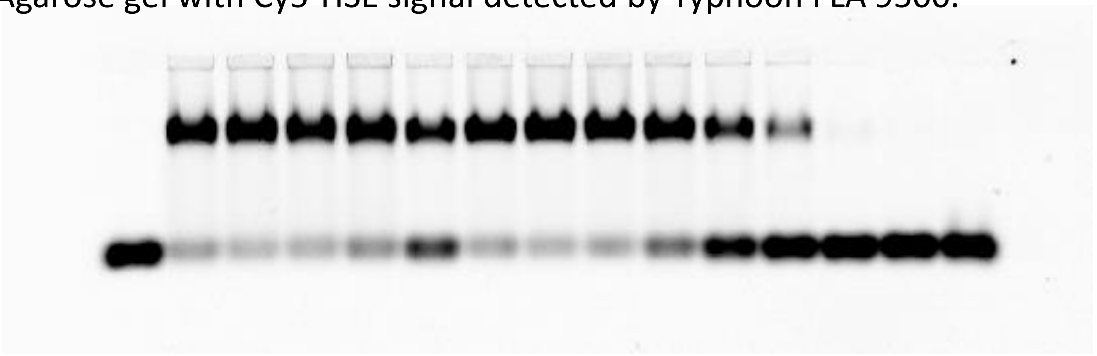

Raw data to figure 4

Blue native electrophoresis of monomeric HSF1 incubated w/wo BSA at 38°C for increasing time, followed by western blotting. Detection by anti HSF1 antibody c-5.

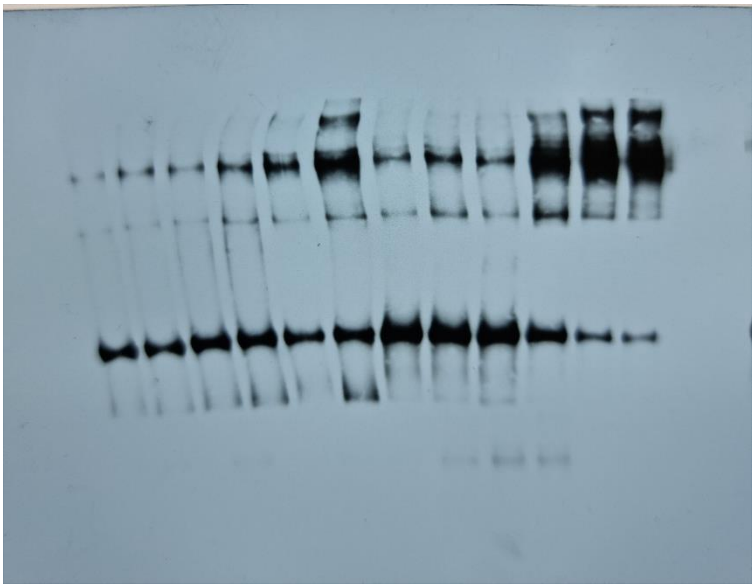

Raw data to figure 4

Blue native electrophoresis of monomeric HSF1 incubated with increasing concentrations of BSA (0-50 mg/ml) at 37°C for 5 minutes, followed by western blotting. Detection by anti HSF1 antibody c-5.

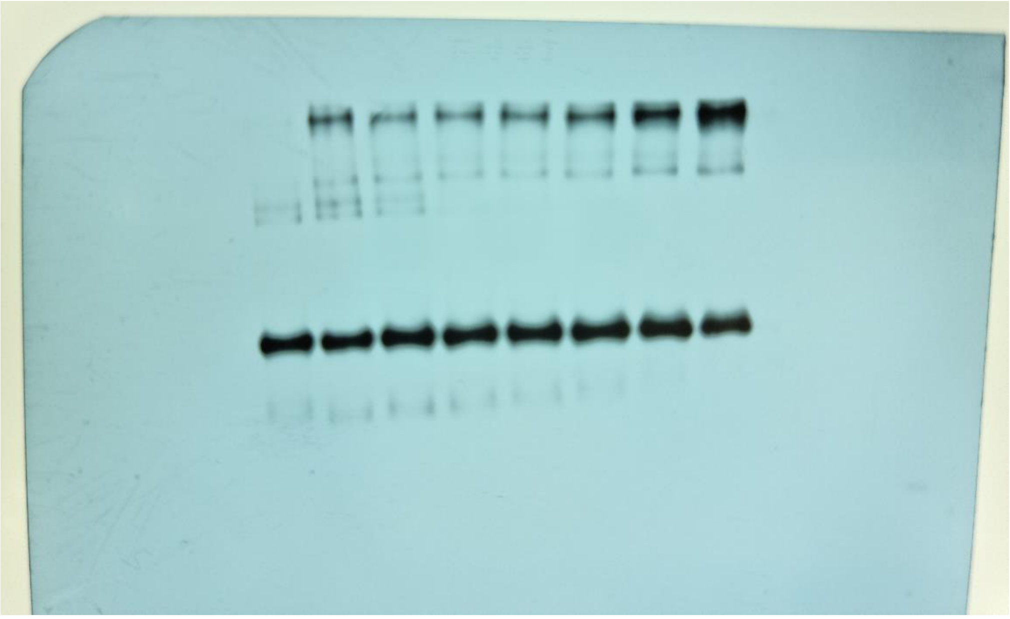

Raw data to figure 4

EMSA using monomeric HSF1 and Cy5-HSE. Samples were incubated w/wo BSA (50 mg/ml) at 39°C for increasing times (0-10 minutes). Agarose gel with Cy5-HSE signal detected by Typhoon FLA 9500.

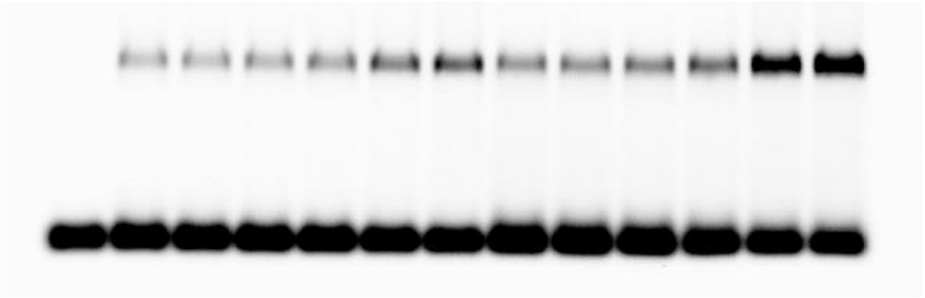

Raw data to figure 4

EMSA using monomeric HSF1 and Cy5-HSE. Samples were incubated with increasing concentrations of BSA (0-100 mg/ml) at 38°C for 5 minutes. Agarose gel with Cy5-HSE signal detected by Typhoon FLA 9500.

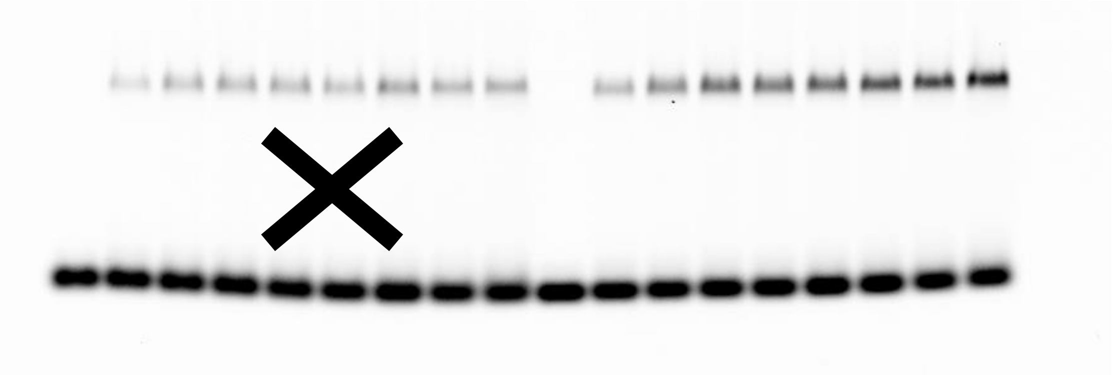

Raw data to figure 4

High resolution clear native electrophoresis. Cells expressing mCherry-HSF1 were incubated with increasing concentrations of sorbitol. Lysates were separated by HR CNE and fluorescence of mCherry-HSF1 was detected in gel by Typhoon FLA 9500.

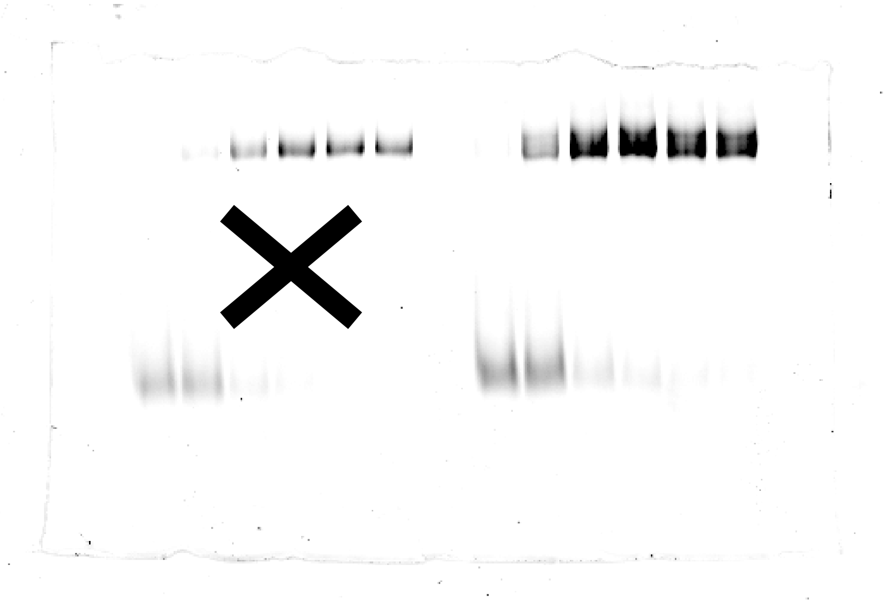

Raw data to figure 5

High resolution clear native electrophoresis. Cells expressing mCherry-HSF1 were exposed to translational inhibitors together with proteotoxic stress. Lysates were separated by HR CNE and fluorescence of mCherry-HSF1 was detected in gel by Typhoon FLA 9500.

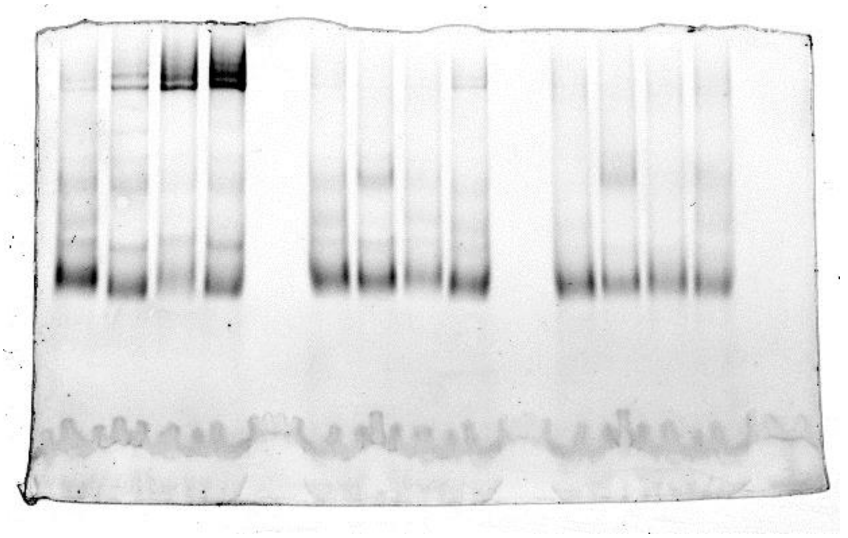

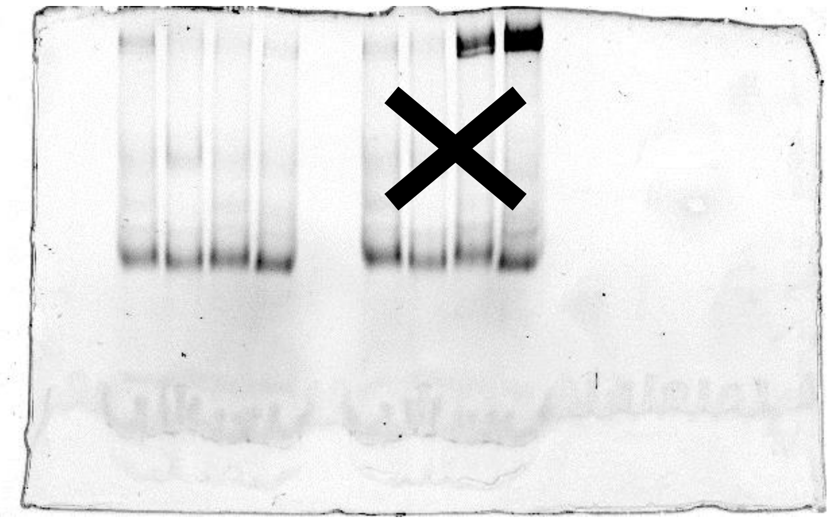

Raw data to figure 5

Chemical crosslinking. Chemically crosslinked HSF1 from cell lysates was analysed by SDS PAGE and western blotting. Detection by anti HSF1 antibody c-5.

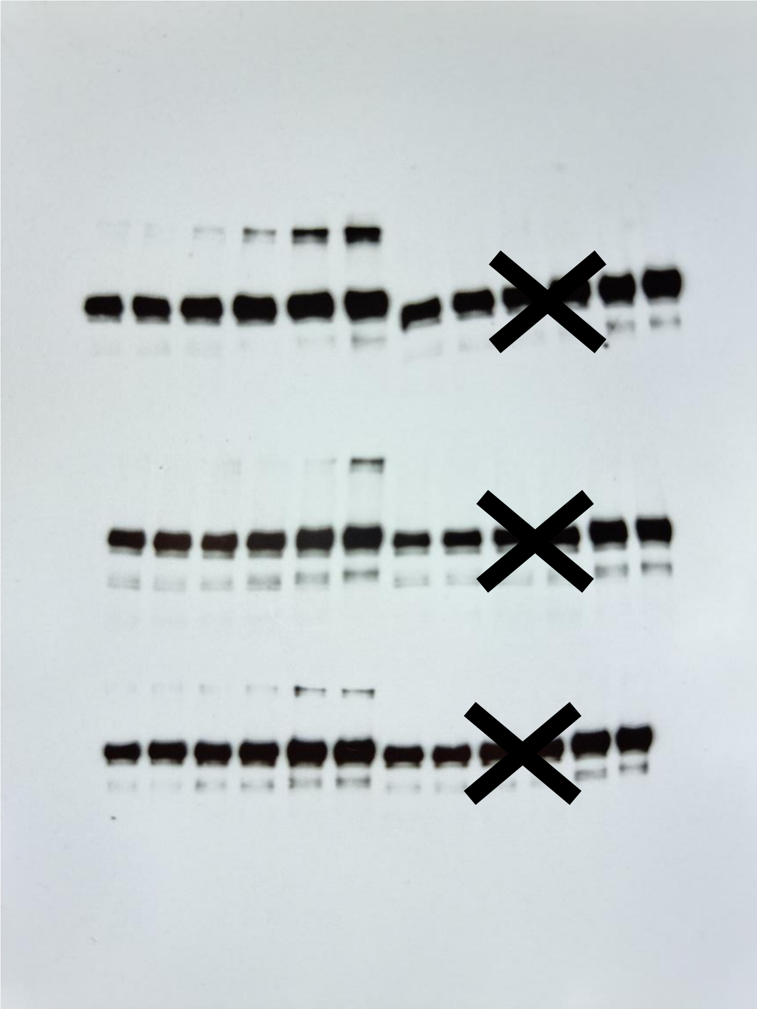

Raw data to figure 5

High resolution clear native electrophoresis. Cells expressing mCherry-HSF1 were exposed to mTOR inhibitors together with AUY-922. Lysates were separated by HR CNE and fluorescence of mCherry-HSF1 was detected in gel by Typhoon FLA 9500.

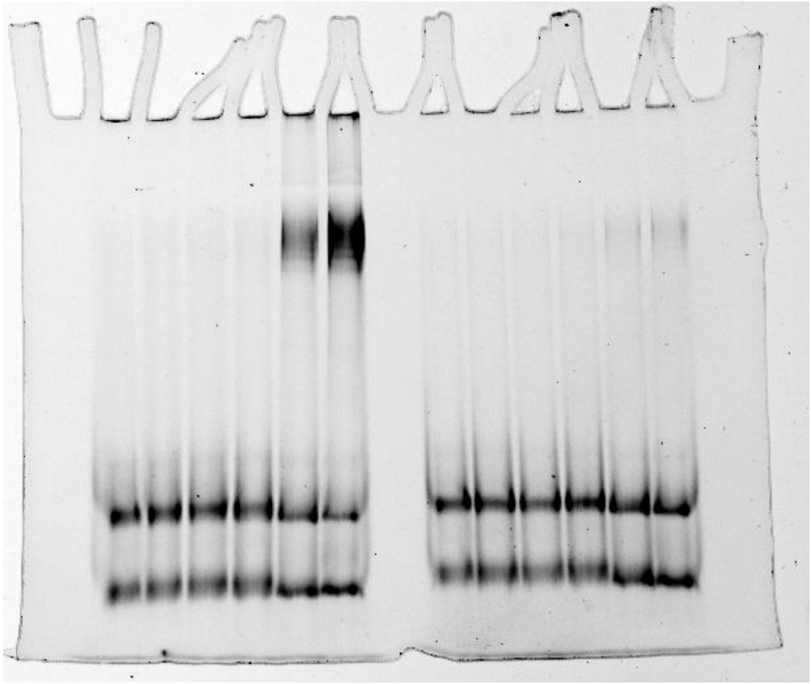

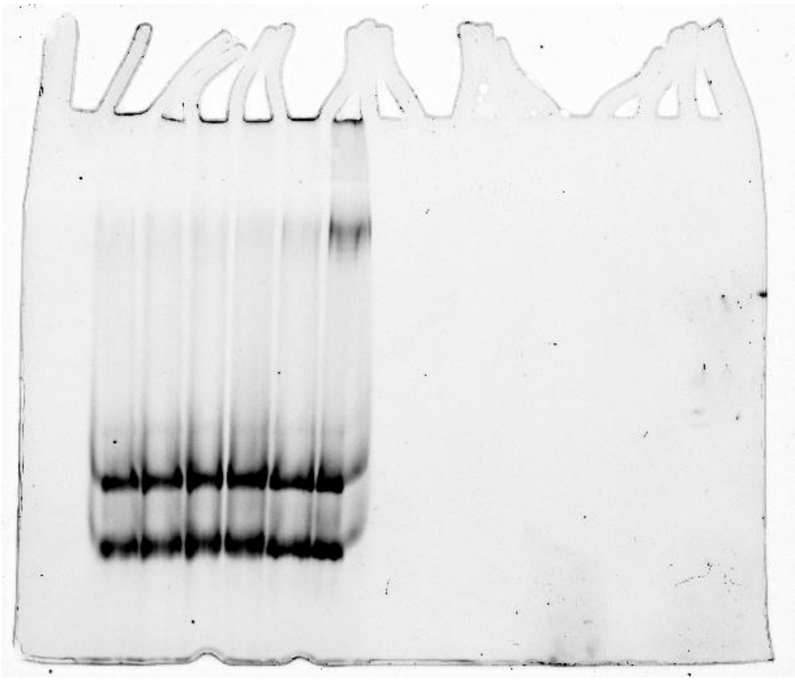

Raw data to figure 6

HSF1 detected by antibody D3L8 in whole cell lysate before pulldown with SBP-Hsp70.

HSF1 detected by antibody D3L8 in whole cell lysate before pulldown with SBP-Hsp90.

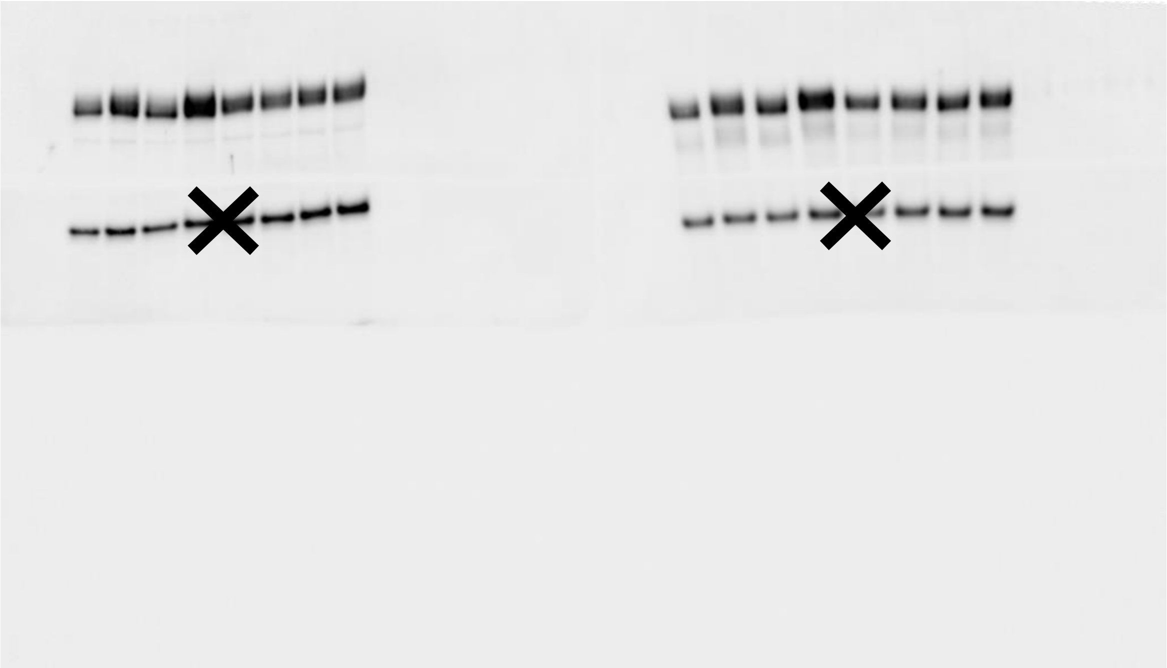

Pulldown SBP-Hsp90 detected by anti HSF1 antibody D3L8 (original whole gel image).

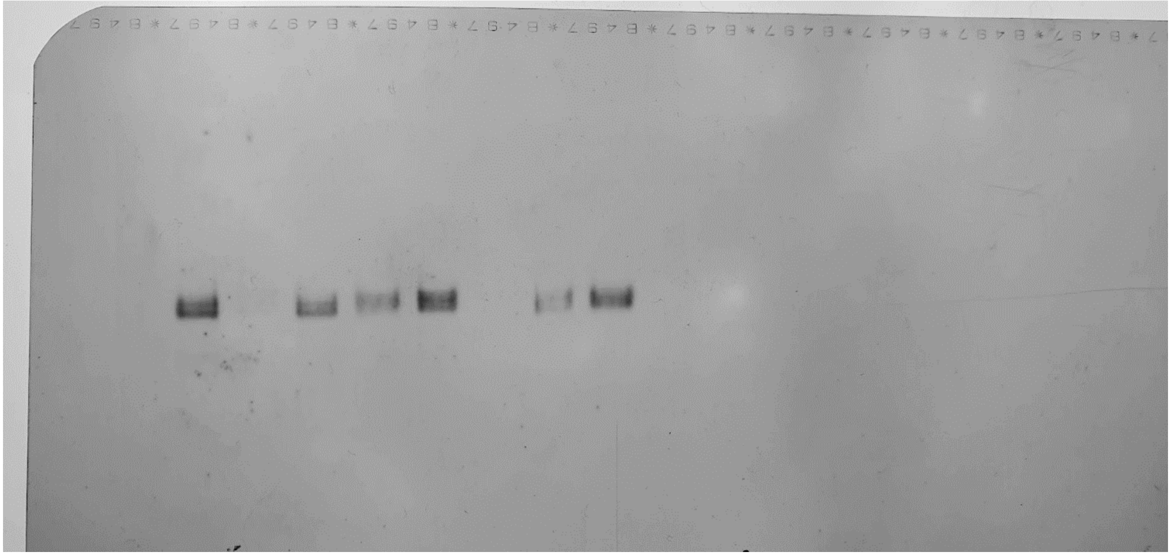

Raw data to figure 6

Pulldown SBP-Hsp70 detected by anti HSF1 antibody D3L8
(image of a western from an independent repetition of the experiment)

Pulldown SBP-Hsp70 detected by anti Hsp70 antibody
(image of a western from an independent repetition of the experiment)

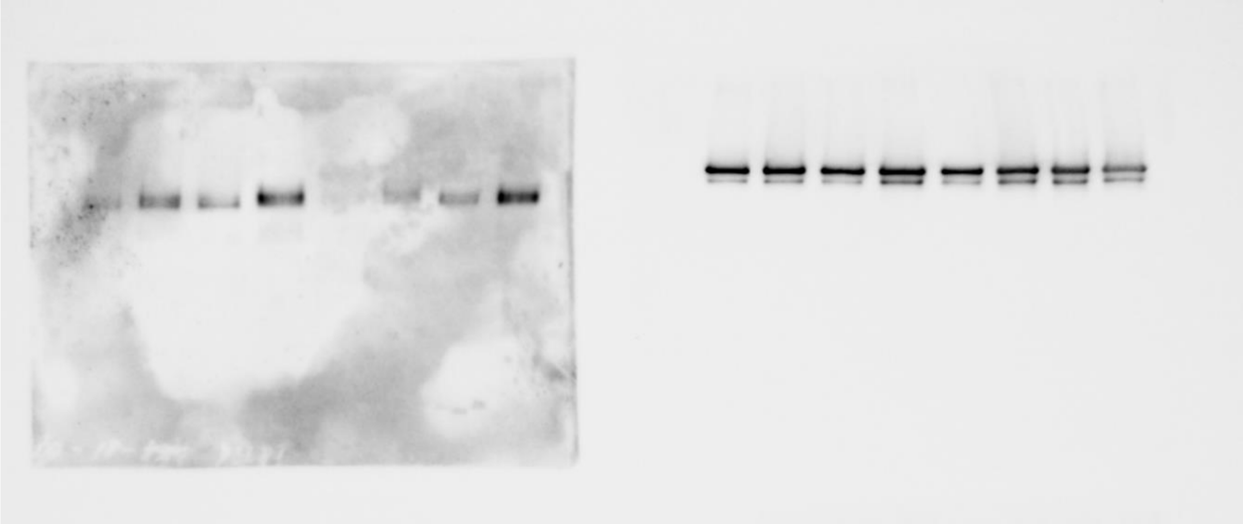

Pulldown SBP-Hsp70 detected by anti HSF1 antibody D3L8
(image of a western from an independent repetition of the experiment)

Pulldown SBP-Hsp70 detected by anti Hsp70 antibody
(image of a western from an independent repetition of the experiment)

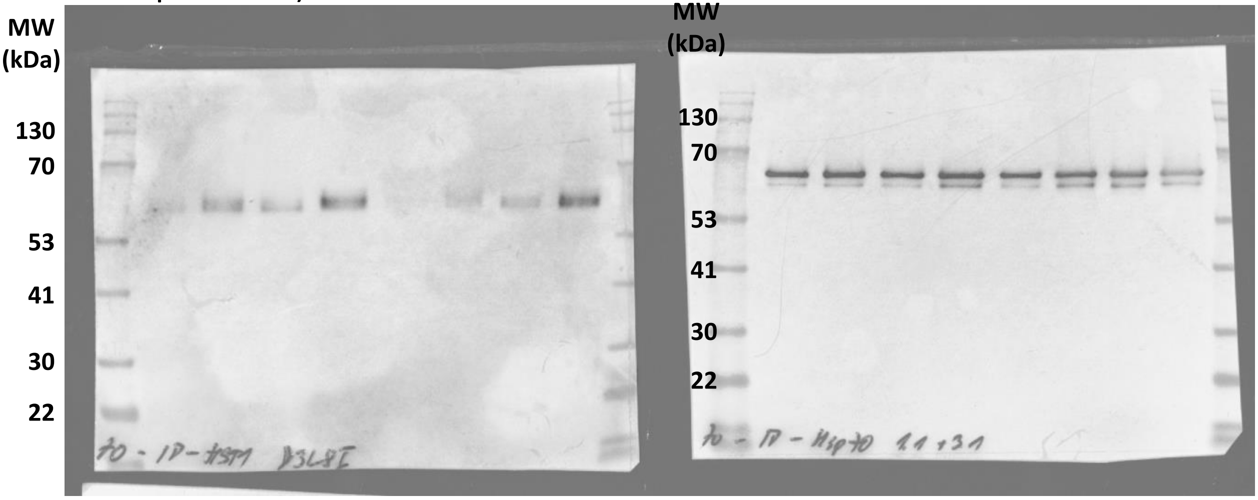

Raw data to figure 6

SBP-Hsp90 pulldown – whole gel

Proteins eluted from streptavidin beads by biotin under native conditions

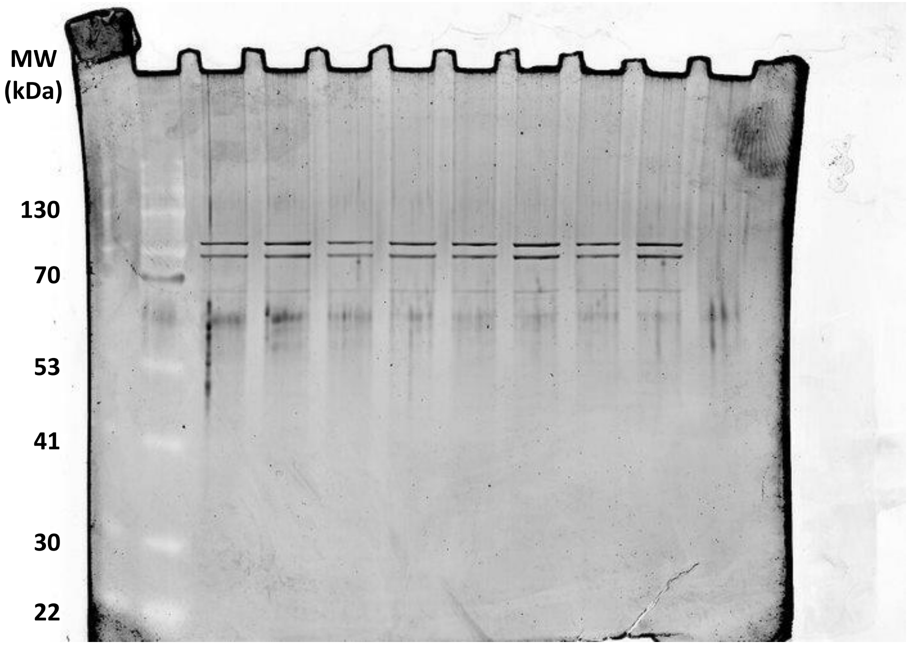

SBP-Hsp70 pulldown –whole gel

Proteins eluted from streptavidin beads by biotin under native conditions

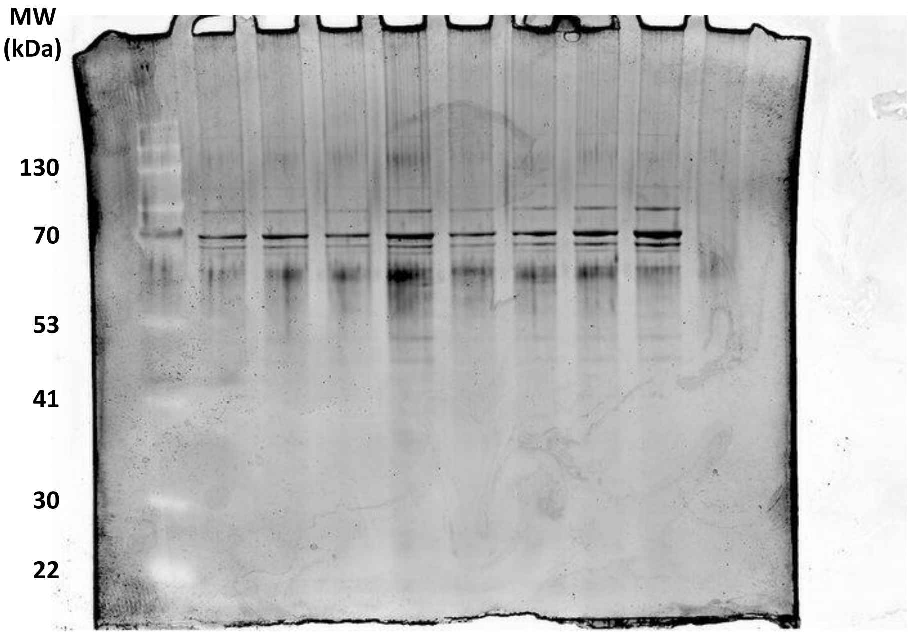

Gels labeled by SYPRO-Ruby and detected by Typhoon FLA 9500.

Raw data to figure S1

SEC fractions separated by SDS PAGE. Whole gel stained by coomassie.

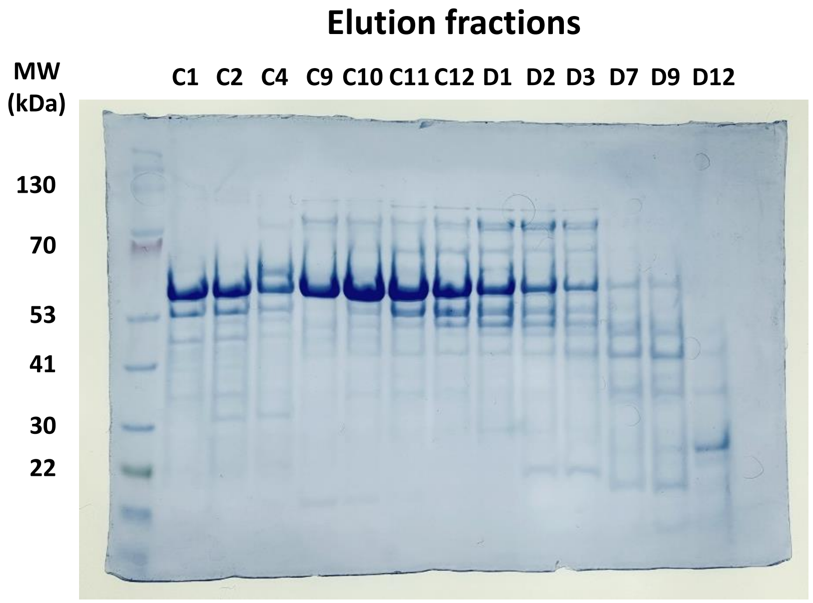

Raw data to figure S3

EMSA using monomeric HSF1 and Cy5-HSE. Samples were incubated with increasing concentrations of BSA (0-100 mg/ml) at 4°C for 30 minutes. Agarose gel with Cy5-HSE signal detected by Typhoon FLA 9500.

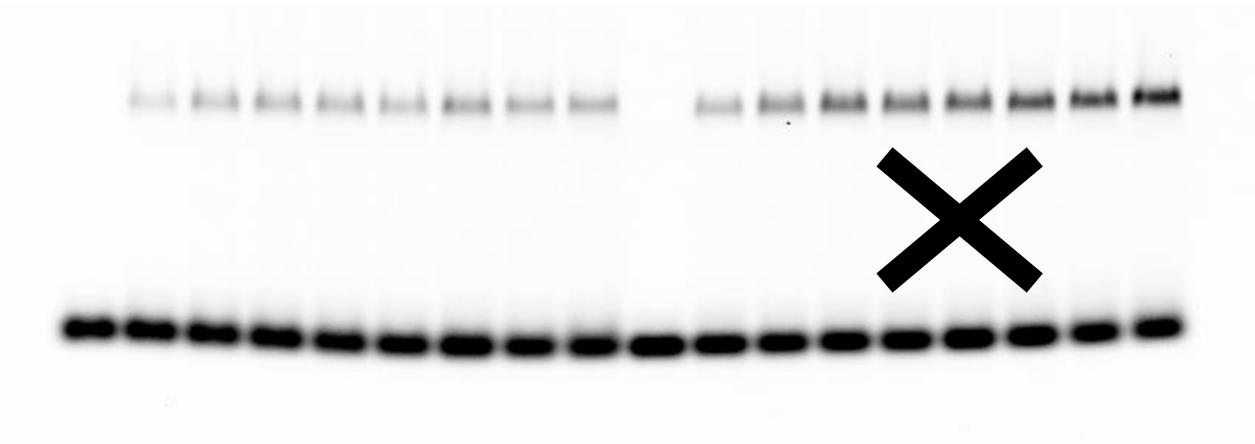

Raw data to figure S4

High resolution clear native electrophoresis. Cells expressing mCherry-HSF1 were incubated with sorbitol for increasing times. Lysates were separated by HR CNE and fluorescence of mCherry-HSF1 was detected in gel by Typhoon FLA 9500.

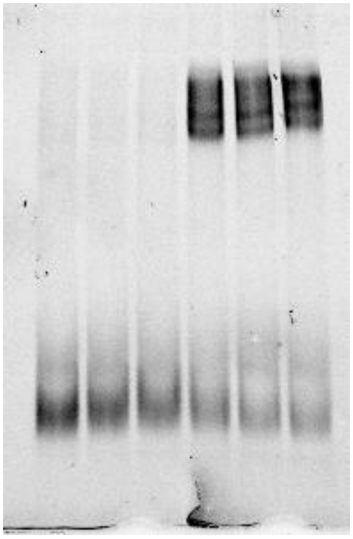

High resolution clear native electrophoresis. Cell lysates containing GFP-HSF1 were incubated with Cy5-HSE and separated by HR CNE. GFP-HSF1 and Cy5-HSE were detected in gel by Typhoon FLA 9500.

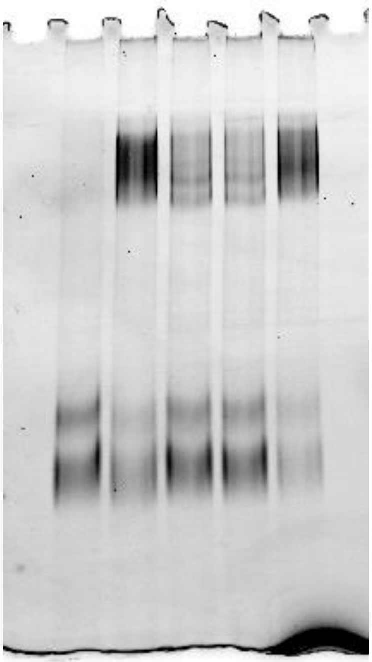

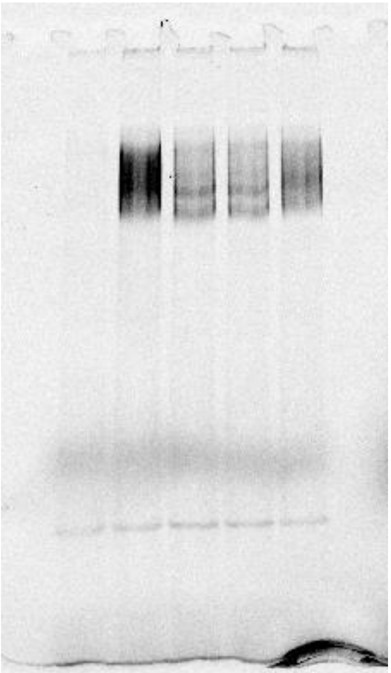

Supplement: S1 Raw images — (PDF) [file pone.0312524.s012.pdf]
