# Supplementary material for: Direct activation of HSF1 by macromolecular crowding and misfolded proteins
Source: PLoS One. 2024 Nov 4;19(11):e0312524. doi: 10.1371/journal.pone.0312524 (PMC11534217; doi:10.1371/journal.pone.0312524)
Supplement: Attachment — Submitted filename: Letter_to_reviewers.pdf [file pone.0312524.s013.pdf]

Masaryk Memorial Cancer Institute

Letter to the Reviewers

PONE-D-24-16853 Direct activation of HSF1 by macromolecular crowding and misfolded proteins PLOS ONE

Dear Reviewers,
We sincerely thank you for your thorough review and valuable feedback on our manuscript. Your insightful comments have been instrumental in improving the quality and clarity of our work. Below, we have addressed each of your comments in detail, outlining the changes made to the manuscript and providing additional explanations where necessary.
We appreciate the opportunity to enhance our research through your constructive critique and look forward to your further evaluation.

Reviewer #1: In this manuscript, the authors investigate the mechanisms underlying the activation of HSF1. Using HSF1 purified from bacteria, the authors show that HSF1 can form 'trimers' on its own at elevated temperature, which leads to an increase in HSF1's DNA binding capacity. The authors claim that in vitro molecular crowding, which mimics the intracellular environment during stress conditions, is the key determinant of HSF1 activation and can occur independent of HSP90 titration. Based on these claims, the authors propose that HSP90-mediated regulation of HSF1 is an indirect consequence of the restoration of proteostasis. While the authors are addressing a fundamentally intriguing question and have performed pertinent experiments, I have several concerns regarding the interpretation of the experiments that warrant the claims of the authors.

Major concerns:
1. The authors have overinterpreted their data in several places to the extent that it appears slovenly. For instance,
a. The authors have equated HSF1 trimerization with cellular activation of HSF1 in many places, including the abstract. HSF1 trimerization, while a key step in the process of HSF1-dependent transcriptional activation of its target genes, is not sufficient to drive activation of the genes.

b. How are the authors certain that the higher molecular weight species in Figs. 2A, 2B, 4A, 4B, 4D and 5 are indeed HSF1 trimers? It is known that HSF1 can form oligomers and aggregates. Therefore, it is unclear if the larger molecular species are HSF1 "trimers", especially when appropriate molecular weight standards are not used or included in the experiments.

c. The authors inhibited protein synthesis and observed a reduction in HSF1 activation, attributing it to the lack of molecular crowding. However, many previous studies have shown that activation of the heat shock response is dependent on the newly synthesized proteins, as they are the key clients of the molecular chaperones. The authors may want to discuss those models as well.

Thank you for your thorough and constructive comments on our manuscript. We have carefully considered your feedback and made significant revisions to address your concerns.
We acknowledge that HSF1 trimerization, while a key step in the process of HSF1-dependent transcriptional activation, is not solely sufficient to drive the activation of target genes. To mitigate any over-interpretation of our data, we have revised the manuscript, including the abstract, to more

accurately reflect this understanding. Additionally, we have conducted further experiments to verify the purity and functionality of HSF1 trimers. Specifically, we utilized electrophoretic mobility shift assays (EMSA) with fluorescent-labeled HSE oligonucleotides to confirm DNA binding ability.

We understand the importance of accurately identifying HSF1 trimers and distinguishing them from oligomers and aggregates. To address this, we performed additional EMSA experiments using Cy5-labeled oligonucleotides with cell lysates. These results, now included in the supporting information files (S4 Fig), demonstrate that the putative trimer can bind to DNA, unlike the monomeric form, thereby excluding the possibility of inactive aggregates. In native gels, we did not use molecular markers because the rate of migration in native electrophoresis with the setup used is affected by charge, conformation, and interaction with the gel phase, in addition to the size of the protein. For this reason, we chose an alternative using fluorescence-labeled oligonucleotides to verify the trimeric DNA binding form of HSF1.
Additionally, to confirm the transactivation capacity of HSF1 under stress conditions, we performed RT-qPCR to assess induction of Hsp70 (HSPA1). Hsp70 mRNA levels correlate well with HSF1 activation, providing further validation of our findings (S4 Fig).
We have revised the manuscript to discuss existing models, including the chaperone titration model, which highlights the role of newly synthesized proteins as key clients of molecular chaperones.

2. In Fig. 2D, how is the concentration of 'trimer' measured with respect to the monomer? In theory, a molar equivalent of a trimer should have 3X more HSF1 molecules than that of a monomer, leading to a much higher protein concentration. The authors must clarify how the concentrations were compared.

Thank you for your insightful comment. The calculation was adjusted to account for the molar amount of both the trimer and the monomer. Specifically, at the same molar concentration, the trimer contains three times the amount of protein compared to the monomer due to its composition of three HSF1 molecules. The trimer fraction was purified using size exclusion chromatography (SEC), ensuring that it exclusively contains the trimeric form without any aggregates or monomer contamination. Similarly, we utilized a fraction of pure monomer. For the monomer sample subjected to heat shock, we assumed that most of the protein would transition to the trimeric form. This assumption is supported by the dissociation constant, which approaches that of the pure trimer. To ensure the quality of the protein, we performed additional experiments, including repeats of SEC, and verified the fractions of the purified protein by dynamic light scattering (DLS). The functional activity of HSF1 was confirmed by electrophoretic mobility shift assay (EMSA) with heat shock elements (HSE), demonstrating that binding in the monomeric fractions to HSE was only detected after heat shock. We have also added a methodological section concerning fluorescence polarization to the manuscript. This section presents the methodology for calculating the dissociation constant and clarifies that we calculated the molar concentration of the whole trimers.

Similarly, heating at 38 ˚C for 10 minutes can lead to oligomerization of HSF1, as seen in Fig. 4A. These oligomers may artifactually alter the tumbling rate of the fluorochrome labeled HSE, resulting in enhanced polarization. The authors may want to incorporate an orthogonal approach such as EMSA, which will also reveal the relative sizes of the free and protein-bound DNA.

Thank you for your valuable feedback regarding the potential effects of oligomerization on the tumbling rate of the fluorochrome-labeled HSE and the suggestion to incorporate an orthogonal approach such as EMSA.

In response, we have supplemented the manuscript with additional experiments using electrophoretic mobility shift assay (EMSA). Specifically, we conducted an experiment in a similar setup to the fluorescence polarization assay using Cy-5 labeled HSE oligonucleotide. Furthermore, EMSA was employed as an independent method to observe the effect of albumin on HSF1 trimerization. Unlike native electrophoresis, this experiment demonstrates that trimer formation occurs and is capable of binding to DNA.
These additional experiments provide a comprehensive validation of our findings, addressing the concerns raised about potential artifacts in the fluorescence polarization measurements and confirming the DNA-binding capability of the HSF1 trimers.

3. In Fig. 4, as the authors used BSA in conjunction with a higher temperature, which might cause HSF1 to oligomerize on its own, the experiment assessing the effects of BSA in Figure 4 is inconclusive. The authors should test the role of BSA at the monomeric temperature of HSF1 used in Fig. 2C.

Thank you for your insightful feedback regarding the potential influence of BSA and temperature on HSF1 oligomerization in Figure 4.
Our experiments indicate that the conversion rate of HSF1 from monomeric to trimeric forms is influenced by both temperature and albumin concentration. The experimental conditions were carefully chosen to minimize the formation of HSF1 aggregates or coaggregates with albumin. To address the specific concern about the role of BSA at a lower temperature, similar to that used in Fig. 2C, we have conducted additional experiments.
To confirm that high concentrations of albumin promote the formation of active HSF1 trimers, we utilized an alternative method, electrophoretic mobility shift assay (EMSA). We have supplemented the manuscript with these new experiments, demonstrating that albumin, as a natural crowder, facilitates the formation of HSF1 trimers capable of binding to DNA across a range of temperatures (37-40°C). This is supported by both native electrophoresis and EMSA.
These additional experiments provide further clarity on the role of albumin and temperature in HSF1 oligomerization and substantiate our findings under varying conditions.

4. In Fig. 4C, the osmotic stress the authors use in their molecular crowding experiments causes a cascade of previously identified cell signaling pathways to be activated. Thus, HSF1 may be activated by mechanisms other than molecular crowding in osmotic stress conditions. The authors must use caution in interpreting their data.

We acknowledge that increased osmotic pressure and cell shrinkage can indeed lead to various cellular changes, including HSF1 activation. However, the primary aim of our experiment was to refute the hypothesis that increased molecular crowding does not lead to HSF1 oligomerization. If our data had shown that increased crowding and cell shrinkage due to osmotic stress did not lead to HSF1 activation, it would have indicated that increased protein crowding alone does not directly activate HSF1. Our findings demonstrate that the results of the osmotic stress experiments align with those of the isolated protein experiments. Therefore, we conclude that excessive crowding is the simplest explanation of HSF1 activation. We have revised the manuscript to reflect this conclusion and included a discussion of possible alternative interpretations of our observations.

Did the authors observe any transcriptional changes in the HSF1 target genes upon inducing osmotic shock? Also, what are the granules in nucleus? HSF1's localization into the granules may not necessarily mean that HSF1 is active.

We are aware that trimerization does not necessarily imply activation of HSF1 to transcribe downstream genes. To address the activation of HSF1 following sorbitol treatment, we performed several additional experiments.
Firstly, we added Cy5-labeled HSE oligonucleotide to native electrophoresis to simultaneously detect trimerization and the ability of HSF1 to bind to DNA. This experiment was conducted in control cells and cells exposed to various stress conditions. The results are presented as supporting information files (S4 Fig) and demonstrate induction of HSF1 DNA binding activity in response to sorbitol as well as other stressors.
Additionally, we performed quantification of Hsp70 (HSPA1A), which correlates with HSF1 activity. In contrast to Hsp90 inhibition (AUY-922), osmotic stress did not increase Hsp70 expression (S4 Fig). This result implicates the side effects of osmotic stress that are known to inhibit transcription. These effects also cause the aberrant formation of HSF1 granules, which are distinct from classical nuclear stress bodies formed after exposure to other stress conditions.
We have included the following sentence in the revised manuscript to clarify these findings: "Although osmotic stress-induced increased crowding leads to trimerization of HSF1 and its binding to DNA, the side effects of these conditions prevent efficient transcription initiation."

5. In Fig. 5C, the authors should include blots of control proteins of the cytoplasmic fraction (e.g., GAPDH) and the nuclear fraction (e.g., histone)

We appreciate your suggestion. We used Tubulin as the control protein for the cytoplasmic fraction and PARP for the nuclear fraction. The results from these western blot analyses have been added to Fig. 5C.

6. The authors claim that their experiments with HSP90 inhibitors in conjugation with translation inhibition calls the chaperone titration model into question. However, as alluded above, the newly synthesized proteins are indeed the key chaperone clients. Also, the HSP90 inhibitor used by the authors not only binds in the ATP pocket of HSP90 but may also alter other cell signaling axes. Combining all of it, the authors have a redundant, non-specific inhibition system that may not necessarily inform the HSP90 mode of action. Indeed, the fact that cytoplasmic restoration of proteostasis contributes to the regulation of HSF1 is far more consistent with their own findings. As is, this reviewer is still not convinced that the data presented by the authors challenge the chaperone titration model.

We appreciate the reviewer's insights. We agree that the chaperone titration model is generally valid in demonstrating that depletion of chaperone capacity leads to physical changes in the cytoplasm, which subsequently activates HSF1 directly. Our paper presents a novel mechanism that differs from the existing chaperone titration model. In our mechanism, chaperones do not directly inhibit HSF1 activation but are essential for maintaining the favorable physical conditions necessary for monomer existence during active proteosynthesis. This is not intended to refute the chaperone titration model but rather to clarify the mechanism through which the negative feedback between HSF1 and chaperones operates.
To enhance readability, we clarify that the chaperone titration model complements the direct activation of HSF1. Specifically, we detail a negative feedback loop between HSF1 and chaperones: under proteotoxic stress, direct activation of HSF1 induces chaperones, which then aid in the degradation or refolding of damaged proteins in a crowded cellular environment. Only when protein homeostasis is re-established can the cytoplasmic conditions allow HSF1 to refold into its monomeric form.
We also acknowledge the concern regarding the specificity of HSP90 inhibitors. To address this, we used submicromolar concentrations of HSP90 inhibitors that are highly selective for HSP90. We assessed the

effect on HSF1 at short intervals, up to 2 hours, to minimize off-target effects. Additionally, we used different structurally unrelated inhibitors of HSP90 (17AAG, AUY922, Radicicol) and observed consistent results across these treatments. This approach ensures that our findings are robust and specifically related to the inhibition of HSP90, supporting our proposed mechanism.

7. Experiments in Fig. 6 and the accompanying text requires a thorough reevaluation. In Fig. 6A, how do authors reconcile the increase in the association of HSF1 and HSP70 following heat shock and the opposite for HSP90? The levels of HSF1 are widely varying in the lysate, which makes quantitative interpretation of the data very challenging.

Thank you for your detailed comments on our experiments in Figure 6.
We are aware that when HSF1 is activated by stress, it can simultaneously be stabilized, leading to varying levels of HSF1 in the lysate. This effect may be further enhanced by post-translational modifications that occur following stress, which can also cause a shift in the HSF1 band. This phenomenon is observed in Figure 6 when stress is not combined with the translation inhibitor cycloheximide. With cycloheximide application, HSF1 levels remain balanced because trimerization does not occur under these conditions.
The resulting experiment provides honest data from live cells, where the change in HSF1 levels cannot be optimized by different loading, as this would also change the levels of SBP-Hsp70 or SBP-Hsp90, which are used as bait in this experiment. Thus, the most appropriate way to quantify the interactions between Hsp70, Hsp90, and HSF1 is to maintain a constant concentration of the bait proteins SBP-Hsp70 and SBP-Hsp90.
To reconcile the increase in the association of HSF1 with Hsp70 following heat shock and the opposite effect for Hsp90, we have made the following changes in the manuscript: "The disruption of the HSF1-Hsp90 interaction following Hsp90 inhibition and translation inhibition suggests that Hsp90 is not directly responsible for maintaining HSF1 in its monomeric form. The increased interaction of HSF1 with Hsp70 after stress is consistent with in vitro experiments showing that Hsp70 preferentially binds to trimeric HSF1, subsequently unzipping its trimeric structure. Conversely, Hsp90 has been shown to be unable to convert trimeric HSF1 into monomers. Our cell-based experiments further indicate that while chaperones interact with HSF1 and facilitate conformational changes, the final conformation and trimerization are determined by the physical conditions in the cytoplasm. Thus, chaperones assist in reaching a state of minimal free energy, with the monomeric or trimeric state being influenced by external physical conditions such as molecular crowding or temperature. This explains the observed increase in HSF1-Hsp70 association following heat shock and the decrease in HSF1-Hsp90 association."
To improve quantifying the interactions between Hsp70, Hsp90, and HSF1, we performed densitometric analysis of HSF1 in both the lysate and the sample after pulldown. The level of HSF1 extracted from the chaperone complex was then normalized to the total level of HSF1 in the lysate. The results of this densitometric analysis have been added to the figure to provide a clearer quantitative interpretation.

8. The experiments in Fig. 7 demonstrate the effect of various HSP90 inhibitors on the intracellular environment, but they provide little insight into how HSF1 is activated. Panels A and B only demonstrate that HSP90 inhibitors increase the amount of misfolded proteins contingent upon protein synthesis. Presence or absence of HSF1 (panel C) seems irrelevant here as the activation of HSF1 occurs downstream of protein misfolding.

In this experiment, our objective was to demonstrate that the increase in impedance correlates with the presence of unfolded proteins, which are responsible for HSF1 activation. The inhibition of protein synthesis prevents the accumulation of unfolded proteins, thus avoiding physical changes in the

cytoplasm that we propose leads to HSF1 activation. However, an alternative interpretation is that HSF1 activation following stress leads to a transcriptional response, inducing proteins that increase impedance (e.g., massive induction of Hsp70).
To investigate this alternative hypothesis, we conducted the experiment in cells where HSF1 and HSF2 were knocked out, ensuring that no HSF1/2-mediated stress-induced transcriptional response occurs after HSP90 inhibition. Therefore, our findings indicate that HSF1 activation is a consequence of the physical changes in the cytoplasm, which we measure as an increase in impedance. This supports our conclusion that HSF1 activation is directly linked to the accumulation of unfolded proteins rather than the transcriptional response induced by HSF1.

Minor concerns:
1. Line 43: The authors discuss on three possible modes of HSF1 activation. The authors may want to include a statement that these three modes are not mutually exclusive as reported in the literature.

We appreciate your suggestion to clarify that these scenarios are not mutually exclusive. In response, we have included the following sentence in the manuscript:
"Rather than being mutually exclusive, these scenarios might complement each other, and our research is focused on uncovering how they integrate to regulate HSF1 activation."

2. It will be helpful for the readers if the authors include the sequence of HSE that was used in their fluorescence polarization assays, as well as the equation used to calculate the dissociation constants.

We have included the sequence used: "5´Cy5-CCCCTTCCCGAATATTCCCCC-3´" Additionally, we have added the equation used to calculate the dissociation constants.

3. In Fig. 3, the authors did not provide any rationale for using 42 ˚C for 20 mins, whereas in most of their other experiments they used ~38 ˚C for 10 mins.

This experiment was conducted at 42 ˚C to ensure that 100% of the monomer protein would be converted to the trimer form. Additionally, we did not use albumin to increase trimerization in this experiment because it would interfere with the mass spectrometry measurements. We have added this rationale to the manuscript text to provide a clearer understanding of our experimental design and conditions.

4. Are these western blots in Figs. 4A and 4B? If not, then how are the authors able to differentiate between albumin and HSF1? An explanation of the methodology will be helpful to the readers.

Yes, the experiments presented in Figures 4A and 4B were conducted using Western blotting. This allows us to specifically differentiate between albumin and HSF1 by using antibodies that target HSF1, ensuring that only the HSF1 protein bands are visualized and analyzed.
We have added this methodological detail to the manuscript text to provide clarity for the readers and ensure a comprehensive understanding of our approach.

Reviewer #2: This study offers intriguing insights into the mechanisms by which the stress responder transcription factor HSF1 is activated. The authors conducted a series of in vitro and in vivo experiments to demonstrate that HSF1 activation is driven by protein crowding which leads to the stress induced conformational changes in HSF1 rather than the previously accepted mechanisms involving chaperone binding or post-translational modifications.

The experiments are well-designed, however, lack proper controls. The following issues need to be addressed.

Figure 2 A, B: The authors show that the peaks in gel filtration correspond to trimeric and monomeric forms of HSF1.To validate that the peaks indeed correspond to trimer and monomer, it is essential to run molecular weight markers alongside the samples. By plotting a standard curve of known molecular weights versus elution volumes, the authors can more accurately determine the molecular weights of the observed peaks. Complementary techniques such as Dynamic Light Scattering (DLS) or Analytical Ultracentrifugation (AUC) should be employed to further substantiate the oligomeric states of HSF1.

We appreciate the opportunity to address your concerns regarding the validation of the peaks in gel filtration chromatography corresponding to the trimeric and monomeric forms of HSF1.
We have employed several independent methods to validate our findings. Using Electrophoretic Mobility Shift Assay (EMSA), we confirmed that the trimeric form of HSF1, unlike the monomer, has the ability to bind to HSE oligonucleotides. Additionally, we demonstrated that temperature shock can induce trimerization and DNA binding in monomeric HSF1. To ensure the purity of each fraction, we conducted SDS gel electrophoresis, which showed that both the monomer and trimer fractions contain HSF1 with high purity.
To further corroborate that the individual peaks on the SEC correspond to the monomeric and trimeric forms of HSF1, we performed parallel separations using BSA and immunoglobulin as molecular weight markers (S1 Fig). In these experiments, the elution of BSA (66.5 kDa) and mouse IgG1 (150 kDa) broadly correlated with the shift between HSF1 monomer (57.26 kDa) and its trimer (171.78 kDa). However, we noted that monomeric HSF1 eluted faster than BSA, and trimeric HSF1 faster than IgG, which we attribute to the inherent characteristics of the size exclusion chromatography technique. Specifically, this method primarily depends on the hydrodynamic radius of the particles. Proteins with unstructured regions, such as HSF1, have a larger hydrodynamic radius compared to globular proteins like BSA and IgG, leading to their faster passage through the column.
To further substantiate that the individual fractions contain monodispersed protein particles in the form of monomers or trimers and that these forms remain stable after freezing and thawing, we assessed the protein quality of selected fractions using Dynamic Light Scattering (DLS). The characterization of the size distribution in the monomer and trimer fractions is included in the supporting information files.
We believe that these comprehensive analyses, including the use of molecular weight markers, EMSA, SDS gel electrophoresis, and DLS, robustly support the identification of the SEC peaks as corresponding to monomeric and trimeric forms of HSF1.

Figure 3: The authors suggest a conformational change in HSF1. To provide stronger evidence of the conformational change in HSF1, the authors should perform Circular Dichroism (CD) spectroscopy, which can detect changes in secondary structure. Additionally, fluorescence spectroscopy, particularly using intrinsic tryptophan fluorescence or extrinsic probes such as ANS (8-Anilino-1-naphthalenesulfonic acid), could be used to monitor tertiary structure changes.

We appreciate your suggestion to include Circular Dichroism (CD) spectroscopy and fluorescence spectroscopy to provide additional evidence of the conformational change in HSF1. While these techniques are valuable for detecting changes in protein structure, we believe that Hydrogen-Deuterium Exchange Mass Spectrometry (HDX-MS) is a more suitable method for our study.

HDX-MS offers several advantages over CD and fluorescence spectroscopy. Firstly, HDX-MS provides higher resolution and specificity in identifying conformational changes at the peptide level, allowing us to pinpoint specific regions of HSF1 that undergo structural alterations. In contrast, CD spectroscopy primarily provides information on overall secondary structure content and may not detect subtle or localized changes. Additionally, fluorescence spectroscopy, while useful for monitoring tertiary structure changes, can be influenced by the environment of the fluorophores and may not accurately reflect conformational changes in all regions of the protein. Moreover, HDX-MS can reveal dynamic structural changes and protein-ligand interactions with high precision, which are critical for understanding the functional mechanisms of HSF1 under different conditions. This method has been successfully used in many studies to investigate protein folding, conformational dynamics, and interactions.

In addition to HDX-MS, we have also performed Electrophoretic Mobility Shift Assay (EMSA) and Size-Exclusion Chromatography (SEC) to address conformational changes in HSF1. EMSA allowed us to observe changes in DNA-binding activity, which can reflect conformational states, while SEC provided insights into the oligomeric state and overall shape of the protein under various conditions. These complementary techniques further support our findings and provide a comprehensive view of the conformational changes in HSF1.

To provide a complete picture of the conformational changes, we have included the results on the deuteration of each identified peptide covering the entire HSF1 sequence. Plots of the time dependence of deuteration of each peptide have been added as supplementary data (Figure S5). For a detailed analysis of the spectra, the primary mass spectrometry data are additionally deposited to the ProteomeXchange Consortium via the PRIDE repository with the dataset identifier "PXDPXD037662"; Username: reviewer_pxd037662@ebi.ac.uk, Password: ChSpggcg.

For these reasons, we have chosen native electrophoresis, HDX-MS, EMSA, and SEC to provide detailed and specific insights into the conformational changes of HSF1. We hope this explanation clarifies our choice of methodologies and their advantages for our study.

References:

Masson, G. R., et al. (2019). "Recommendations for performing, interpreting and reporting hydrogen-deuterium exchange mass spectrometry (HDX-MS) experiments." Nature Methods, 16(7), 595-602.

Englander, S. W., & Mayne, L. (2014). "The case for defined protein folding pathways." Proceedings of the National Academy of Sciences, 111(45), 15873-15880.

Rand, K. D., & Zehl, M. (2019). "Hydrogen/deuterium exchange mass spectrometry for structural biology: is there a strategy for 'middle-down'?" Expert Review of Proteomics, 16(5), 367-373.

Wales, T. E., & Engen, J. R. (2006). "Hydrogen exchange mass spectrometry for the analysis of protein dynamics." Mass Spectrometry Reviews, 25(1), 158-170.

Figure 4 A, B: The trimerization of HSF1 in the presence of 50 mg/mL BSA at 38ºC (Fig 4A) and at 37ºC (Fig 4B) shows different extents of trimerization, with a higher amount of monomer present at 37ºC. The discrepancy between the trimerization extents at 37ºC and 38ºC needs to be addressed. The authors should perform additional replicates and statistical analyses to determine if the observed differences are significant and reproducible. Detailed information about the experimental conditions, such as precise temperature control, sample preparation, and timing, should be provided to rule out technical variability. Exploring the effects of incremental temperature changes on trimerization could also be informative. Discussing possible biochemical or biophysical reasons for the observed anomaly in trimerization extent would be valuable. This could include differences in protein stability, aggregation propensity, or interactions with BSA at slightly different temperatures.

Figure 4A. The trimerization of purified Hsf1 increases with time till 600 sec, whereas monomeric Hsf1 decreased with time only in presence of BSA. The author should explain the discrepancy of the observation.

Thank you for your detailed and constructive feedback on Figure 4.
Our goal was to demonstrate that both albumin-induced crowding and elevated temperature influence the rate and extent of HSF1 trimerization. We acknowledge that the Western blot images may not fully capture the quantitative differences between monomer and trimer due to the nature of the technique, where factors such as the length of exposure can significantly affect the visibility of subtle differences. Our intention was to present the results in their most natural form to ensure that all bands were visible.
To address the concerns about the differences in trimerization extents at 37ºC and 38ºC, we repeated the experiment to ensure the reproducibility of our findings. Detailed information about the experimental conditions, including precise temperature control, sample preparation, and timing, has been provided to rule out technical variability.
Furthermore, to independently confirm that increasing albumin concentration positively affects HSF1 trimerization by activating it to bind to DNA, we conducted a similar experiment using EMSA. This method accurately reflects the amount of trimer binding to the fluorescently labeled oligonucleotide HSE. The results of these EMSA experiments have now been included in Figure 4.
To address the request for a discussion on the possible biochemical or biophysical reasons for the observed anomaly in trimerization extent, we have added the following information to the discussion:
"When interpreting the effect of crowding on HSF1, it should be taken into account that its activation does not consist only of trimer assembly but involves the breakdown of the monomeric conformation, which is followed by trimer assembly through the hydrophobic HR-A/B regions. Based on studies exploring the impact of protein crowders on protein conformation, we propose that both soft attractions and hard sphere repulsions may contribute to the conformational switch and trimerization of HSF1. Furthermore, another factor that potentiates trimerization along with crowding is increased temperature. Studies on the effects of temperature and hydrostatic pressure on the dynamical properties and folding stability of highly concentrated lysozyme solutions revealed that elevated temperatures can lead to a destabilization of the protein structure due to transient destabilizing intermolecular interactions. This effect is particularly noticeable under conditions at very high protein concentrations, which mimic densely packed intracellular environments https://doi.org/10.1039/C7CP00705A ."
These insights highlight the complex interplay between crowding effects and temperature on protein conformational changes and stability, suggesting that the observed anomaly in HSF1 trimerization extent could be due to these factors influencing its conformational dynamics and interaction propensity under varying conditions.
We hope these additional experiments and detailed explanations address your concerns and provide a clearer understanding of our findings.

Figure 4 C, D: The results suggest that macromolecular crowding and misfolded proteins activate HSF1, and the interpretation that HSF1 activation is independent of chaperone interaction needs further validation. Supplement the qualitative observations with quantitative data showing the extent of HSF1 activation under various crowding conditions. The choice of a 20-minute duration for observing crowding effects should be justified. It would be beneficial to explore the time-dependence of HSF1 activation by conducting a time-course experiment, assessing activation at multiple time points to understand the kinetics of the response. To robustly conclude that HSF1 activation is independent of chaperone interaction, additional experiments should be performed.

Figures 4C and Fig 5B: The authors should show the nuclear localization of mCherry-HSF1 with nuclear staining dye.

Thank you for this valuable feedback. We have addressed your comments as follows:
Nuclear localization of mCherry-HSF1: This experiment utilized live cell imaging of mCherry-HSF1, where the mCherry-HSF1 signal accurately represents the nucleus. Therefore, initial images did not include nuclear staining. To confirm HSF1 localization in the nucleus, we have stained control cells with Hoechst. The colocalization of mCherry-HSF1 with Hoechst-stained nuclei is clear in these images, now included as supplementary data (S2 Fig).
We have conducted additional experiments to validate that sorbitol-induced crowding leads to HSF1 trimerization and DNA binding. We analyzed the time-dependence of sorbitol's effect on cells using native electrophoresis to evaluate HSF1 trimerization. Additionally, we included a Cy5-labeled HSE oligonucleotide in the native electrophoresis to simultaneously assess HSF1 trimerization and DNA binding capacity. These additional results are provided in supplementary data (Figure S4).

Figure 5C. The western blot data is missing controls. A marker protein for cytosol and nucleus should be included in the blot. A significant amount of Hsf1 is found in the nucleus (Fig 5D) in presence of drug upon heat shock which is not found in Fig. 5C. Also, the distribution of Hsf1 in cytosol and nucleus in control and stress condition is not proportional.

In response, we have added Western blots for tubulin as a cytoplasmic marker and PARP as a nuclear marker. The discrepancy between the amount of HSF1 in the nuclear and cytoplasmic fractions can be attributed to a different methodological approach. The experiment in Figure 5D used cells stably expressing mCherry-HSF1, which have higher levels of HSF1 compared to endogenous HSF1. This may result in slightly different behavior during fractionation. To address this issue and provide more accurate data, we performed a similar experiment on a parental cell line with endogenous HSF1. In this experiment, HSF1 was detected using a specific antibody, increasing the specificity of detection compared to total fluorescence measurements and eliminating other possible artifacts.
For clarification, we have added the following information to the text: "To exclude artifacts associated with fluorescent labeling, we conducted cell fractionation experiments using H1299 cells containing endogenous HSF1, subjected to the same conditions as in previous experiments."

Figure 6 A, B. The western blots of the pull-down assay lack molecular weight markers. The loading control is missing in cell lysate. What is the negative control for this experiment? Only bead without overexpressing Hsp70/Hsp90 cell lysate is essential in the western blot data. The full blot of immunoprecipitated Hsf1 is required to assess the monomeric or trimeric form of Hsf1 bound to chaperone.
According to the chaperone hypothesis binding of chaperones with Hsf1 will be reduced upon application of stress. However, immunoprecipitation of Hsp70/Hsp90 with Hsf1 suggests no alteration of chaperone binding. In contrast, Hsp70 binding to monomeric Hsf1 is enhanced upon heat stress. The western blot data showed induction of Hsf1 upon heat stress although no chaperone induction was found. Have the authors tried to see the binding of trimeric Hsf1 with chaperones in normal and stress conditions. Moreover, what will happen if purified Hsp70 is incubated with purified Hsf1 and see the transition of monomeric to trimeric conformation of Hsf1 upon stress.

We appreciate your feedback and have addressed the concerns as follows:

Masaryk Memorial Cancer Institute

This experiment was performed with cells stably expressing SBP-Hsp70 or SBP-Hsp90. Pulldown is subsequently performed on a streptavidin matrix from which, after washing, the complexes are eluted with biotin under native conditions to avoid eluting non-specifically bound proteins. This is clearly seen in the gel showing total proteins eluted from streptavidin, with SBP-Hsp70 and SBP-Hsp90 forming the dominant band. To control for nonspecific binding of proteins to the matrix, the experiment used beads in which streptavidin was blocked with biotin beforehand. In this case, there was no binding of SBP-Hsp70 and Hsp90, and no proteins were eluted on addition of biotin. The result from this control experiment is a clear gel, which we felt was redundant to add to the results. We believe that using specifically treated samples as a negative control was appropriate to compare chaperone and HSF1 binding in this experiment: While the interaction of Hsp70 with HSF1 is minimal in control cells, the interaction increases after stress. To assess the interaction between Hsp90 and HSF1, we used cells treated with an Hsp90 inhibitor as a negative control, which leads to a complete disruption of HSF1 binding to Hsp90. Regarding chaperone levels, SBP-Hsp70 and SBP-Hsp90 do not change during treatment because they are expressed from the constitutively activated EF1a promoter. Although the levels of SBP-Hsp90 and SBP-Hsp70 are balanced in the lysate, there is more total protein captured in samples exposed to stress after SBP-Hsp70 pulldown due to increased complex formation with Hsp70 after stress. The proteins in this experiment were analyzed by SDS-PAGE, where denaturation makes it impossible to distinguish monomers from trimers. However, the parallel experiment presented in the previous figures shows that translation inhibitors prevent the formation of trimers. It is evident from the figure showing the total HSF1 levels by SDS-PAGE that a band shift of HSF1 occurs in samples exposed to stress, which can be attributed to post-translational modifications that occur in association with HSF1 trimerization. In contrast, the shift does not occur when translation inhibition is present to prevent trimerization.

We have appended full blots, including controls, to the supplementary data of the revised manuscript. We also performed densitometry of HSF1 in lysate and HSF1 detected in complex with chaperones. We then normalized the HSF1pulldown level to the total HSF1 level, which is more stable due to stress and trimerization.

Experiments focusing on the interactions between purified HSF1 monomer or trimer would be of great interest, but are beyond the scope of the present paper. The interaction between the chaperones Hsp70 and Hsp90 has been addressed in detail in recent papers, which, in agreement with our findings, have discussed the interaction between Hsp70 and HSF1 trimer.

Kmiecik SW, Le Breton L, Mayer MP. Feedback regulation of heat shock factor 1 (Hsf1) activity by Hsp70-mediated trimer unzipping and dissociation from DNA. EMBO J. 2020 Jul 15;39(14):e104096. doi: 10.15252/embj.2019104096. Epub 2020 Jun 3. PMID: 32490574; PMCID: PMC7360973.

We appreciate your constructive feedback, and we hope that the additional data and text clarifications have strengthened the findings of and improved the understanding of our manuscript.

Yours sincerely,

Petr Muller

Brno, July 29th, 2024

Masaryk Memorial Cancer Institute, Zluty kopec 7, 65653 Brno, Czech Republic, muller@mou.cz

Masaryk Memorial Cancer Institute, Zluty kopec 7, 65653 Brno, Czech Republic, muller@mou.cz
